# METTL14 is a chromatin regulator independent of its RNA *N*^*6*^-methyladenosine methyltransferase activity

**DOI:** 10.1093/procel/pwad009

**Published:** 2023-02-23

**Authors:** Xiaoyang Dou, Lulu Huang, Yu Xiao, Chang Liu, Yini Li, Xinning Zhang, Lishan Yu, Ran Zhao, Lei Yang, Chuan Chen, Xianbin Yu, Boyang Gao, Meijie Qi, Yawei Gao, Bin Shen, Shuying Sun, Chuan He, Jun Liu

**Affiliations:** Department of Chemistry and Institute for Biophysical Dynamics, University of Chicago, Chicago, IL 60637, USA; Howard Hughes Medical Institute, Chicago, IL 60637, USA; State Key Laboratory of Protein and Plant Gene Research, Peking-Tsinghua Center for Life Sciences, School of Life Sciences, Peking University, Beijing 100871, China; Department of Chemistry and Institute for Biophysical Dynamics, University of Chicago, Chicago, IL 60637, USA; Howard Hughes Medical Institute, Chicago, IL 60637, USA; Department of Chemistry and Institute for Biophysical Dynamics, University of Chicago, Chicago, IL 60637, USA; Howard Hughes Medical Institute, Chicago, IL 60637, USA; Department of Physiology and Brain Science Institute, Johns Hopkins University School of Medicine, Baltimore, MD 21205, USA; State Key Laboratory of Protein and Plant Gene Research, Peking-Tsinghua Center for Life Sciences, School of Life Sciences, Peking University, Beijing 100871, China; State Key Laboratory of Protein and Plant Gene Research, Peking-Tsinghua Center for Life Sciences, School of Life Sciences, Peking University, Beijing 100871, China; State Key Laboratory of Protein and Plant Gene Research, Peking-Tsinghua Center for Life Sciences, School of Life Sciences, Peking University, Beijing 100871, China; Institute for Regenerative Medicine, Shanghai East Hospital, Shanghai Key Laboratory of Signaling and Disease Research, Frontier Science Center for Stem Cell Research, School of Life Sciences and Technology, Tongji University, Shanghai 200120, China; The Institute of Translational Medicine, School of Medicine, Zhejiang University, Hangzhou 310029, China; Department of Chemistry and Institute for Biophysical Dynamics, University of Chicago, Chicago, IL 60637, USA; Howard Hughes Medical Institute, Chicago, IL 60637, USA; Department of Chemistry and Institute for Biophysical Dynamics, University of Chicago, Chicago, IL 60637, USA; Howard Hughes Medical Institute, Chicago, IL 60637, USA; Division of Life Sciences and Medicine, Center for Reproductive Medicine, The First Affiliated Hospital of USTC, University of Science and Technology of China, Hefei 230000, China; Clinical and Translational Research Center of Shanghai First Maternity & Infant Hospital, Shanghai Key Laboratory of Signaling and Disease Research, Frontier Science Center for Stem Cell Research, School of Life Sciences and Technology, Tongji University, Shanghai 200092, China; State Key Laboratory of Reproductive Medicine, Center for Global Health, Gusu School, Women’s Hospital of Nanjing Medical University, Nanjing Maternity and Child Health Care Hospital, Nanjing Medical University, Nanjing, 211166, China; Department of Physiology and Brain Science Institute, Johns Hopkins University School of Medicine, Baltimore, MD 21205, USA; Department of Chemistry and Institute for Biophysical Dynamics, University of Chicago, Chicago, IL 60637, USA; Howard Hughes Medical Institute, Chicago, IL 60637, USA; Department of Biochemistry and Molecular Biology, University of Chicago, Chicago, IL 60637, USA; State Key Laboratory of Protein and Plant Gene Research, Peking-Tsinghua Center for Life Sciences, School of Life Sciences, Peking University, Beijing 100871, China

**Keywords:** METTL14, chromatin, H3K27me3, m^6^A-independent, mESC differentiation

## Abstract

METTL3 and METTL14 are two components that form the core heterodimer of the main RNA m^6^A methyltransferase complex (MTC) that installs m^6^A. Surprisingly, depletion of METTL3 or METTL14 displayed distinct effects on stemness maintenance of mouse embryonic stem cell (mESC). While comparable global hypo-methylation in RNA m^6^A was observed in *Mettl3* or *Mettl14* knockout mESCs, respectively. *Mettl14* knockout led to a globally decreased nascent RNA synthesis, whereas *Mettl3* depletion resulted in transcription upregulation, suggesting that METTL14 might possess an m^6^A-independent role in gene regulation. We found that METTL14 colocalizes with the repressive H3K27me3 modification. Mechanistically, METTL14, but not METTL3, binds H3K27me3 and recruits KDM6B to induce H3K27me3 demethylation independent of METTL3. Depletion of METTL14 thus led to a global increase in H3K27me3 level along with a global gene suppression. The effects of METTL14 on regulation of H3K27me3 is essential for the transition from self-renewal to differentiation of mESCs. This work reveals a regulatory mechanism on heterochromatin by METTL14 in a manner distinct from METTL3 and independently of m^6^A, and critically impacts transcriptional regulation, stemness maintenance, and differentiation of mESCs.

## Introduction

Among all known internal RNA modifications, *N*^*6*^-methyladenosine (m^6^A) is the most prevalent one on mammalian messenger RNA (mRNA). m^6^A is installed by a large multicomponent methyltransferase complex (MTC, writers), which consists of METTL3 and METTL14 as the core and other regulatory subunits including WTAP (Zhong et al., 2008; Ping et al., 2014), VIRMA (Yue et al., 2018), ZC3H13 (Wen et al., 2018), and RBM15/RBM15B (Patil et al., 2016). m^6^A is known as a reversible modification upon the discovery of two demethylases FTO and ALKBH5 (Jia et al., 2011; Zheng et al., 2013). m^6^A methylated transcripts are regulated by reader proteins during almost all steps of mRNA metabolism, including pre-mRNA processing (Zhou et al., 2019), degradation (Wang et al., 2014), and translation (Wang et al., 2015). The m^6^A methylation on chromatin-associated regulatory RNA (carRNA) critically impacts chromatin state and transcription (Huang et al., 2019; Liu et al., 2020, 2021a; Xu et al., 2021). m^6^A-dependent mRNA regulation is essential in mammals, and its dysregulation affects diverse physiological processes (Frye et al., 2018; He and He, 2021).

As the core MTC components, METTL3 and METTL14 form a stable heterodimer complex (Liu et al., 2014; Wang et al., 2016a). METTL3 is the catalytically active monomer, and METTL14 plays a structural role in RNA substrates recognition. As both METTL3 and METTL14 are required for RNA m^6^A methylation, perturbations of METTL3 and METTL14 are supposed to induce similar defects, or METTL3 depletion would cause a more severe phenotype than METTL14 depletion. However, knockdown of METTL14 had a higher incidence of tumorigenicity in glioblastoma stem cells (GSCs) than knockdown of METTL3 (Cui et al., 2017), suggesting that METTL14 may have other functions in addition to forming a heterodimer with METTL3 to install m^6^A. Recent studies suggest that METTL3 and METTL14 could be recruited to different genomic loci by various chromatin-binding proteins or histone modifiers. For example, both METTL3 and METTL14 were found associated with chromatin fractions; however, they do not bind to the same genomic regions. Only METTL3, but not METTL14, could be recruited to gene promoters through CEBPZ protein in human AML cells (Barbieri et al., 2017). In addition, H3K36me3 facilitates the binding of the m^6^A MTC to adjacent RNA polymerase II to install m^6^A co-transcriptionally through directly binding with METTL14 rather than METTL3 (Huang et al., 2019). These findings suggest that METTL3 and METTL14 may have distinct protein binding partners; these interactions could help recruit the MTC to different genomic loci for m^6^A installation. Besides the potentially different regulatory effects of METTL3 and METTL14 on RNA modification, METTL3 was found to promote translation independently of its methyltransferase activity (Lin et al., 2016). The m^6^A-independent regulatory role of METTL14 has not been well studied.

Here we show that METTL14, but not METTL3, binds H3K27me3 and recruits KDM6B to METTL14-occupied genomic regions in an RNA- and m^6^A-independent manner. In this way, knockout of *Mettl14* impaired the recruitment of KDM6B and led to increased H3K27me3 predominantly at genomic regions bound by METTL14 in mESCs, which repressed downstream gene expression. We further demonstrate that the group of genes regulated by METTL14 at genomic regions co-occupied by METTL14 and H3K27me3 are essential to the self-renewal and differentiation potency of mESCs. Our results suggest that METTL14 regulates heterochromatin in a METTL3- and m^6^A-independent manner, and such a chromatin-level regulation is critical to transcription regulation, stemness maintenance, and mESC differentiation.

## Results

### *Mettl3* and *Mettl14* showed different effects on stemness maintenance and transcription regulation in mESCs

**Figure 1.**
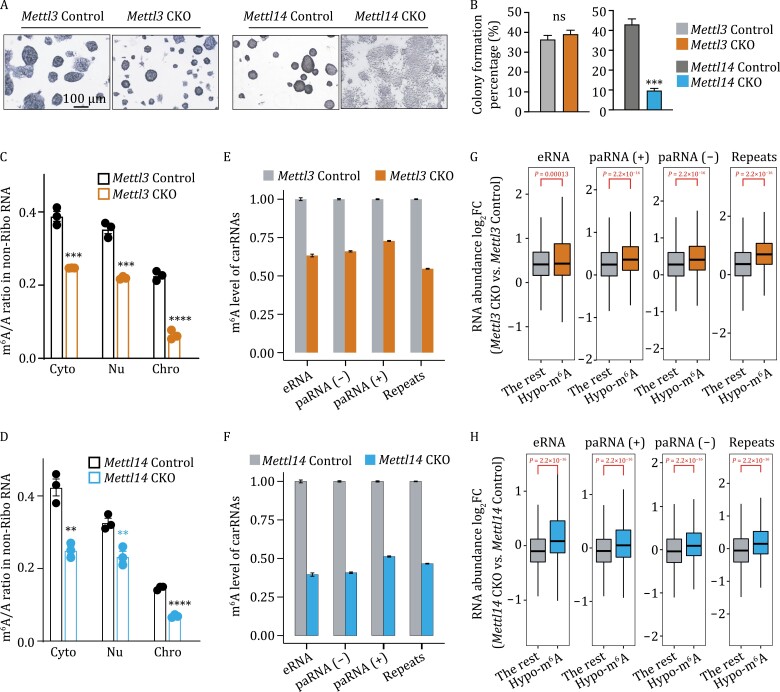
**METTL3 and METTL14 showed different effects on stemness maintenance of mESCs.** (A) Alkaline phosphatase staining of *Mettl3* Control and *Mettl3* CKO, and *Mettl14* Control and *Mettl14* CKO mESCs. (B) Colony formation abilities of *Mettl3* Control and *Mettl3* CKO, *Mettl14* Control and *Mettl14* CKO quantified by AP staining. *n* = 4 biological replicates; error bars indicate means ± SEM. (C and D) LC–MS/MS quantification of the m^6^A/A ratio of the nonribosomal (non-Ribo) RNA in soluble cytoplasmic (Cyto), nucleoplasmic (Nu), and chromosome-associated (Chro) fractions extracted from *Mettl3* Control and *Mettl3* CKO (C), *Mettl14* Control and *Mettl14* CKO (D) mESCs, respectively. *n* = 3 biological replicates; error bars indicate means ± SEM. (E and F) m^6^A level changes on carRNAs were quantified through normalizing m^6^A sequencing results with spike-in in *Mettl3* Control and *Mettl3* CKO (E), *Mettl14* Control and *Mettl14* CKO (F) mESCs, respectively. *n* = 2 biological replicates; error bars indicate means ± SEM. (G and H) carRNAs were divided into hypo-methylated (Hypo-m^6^A) and non-hypo-methylated (The rest) groups in mESCs. Boxplot showing greater increases in RNA abundance fold-changes (log_2_FC) of *Mettl3* CKO vs. *Mettl3* Control (G) or *Mettl14* CKO vs. *Mettl14* Control (H) in the hypo-m^6^A group compared with the rest group in mESCs. *P* values were calculated by a nonparametric Wilcoxon-Mann–Whitney test.

To investigate potential diverse roles of METTL3 and METTL14, we constructed conditional knockout (CKO) of *Mettl3* and *Mettl14* mESCs (Lin et al., 2017), respectively (Fig. S1A and S1B). Both CKO cell lines did show a reduction in cell proliferation (Fig. S1C). To our surprise, the two proteins exerted different regulatory roles in stem cell maintenance: *Mettl3* knockout retained round and compact mESC colony morphology, whereas *Mettl14* knockout could barely support mESC maintenance (Figs. 1A, 1B and S1D), indicating the potential distinct role of METTL14 from METTL3.

As both METTL3 and METTL14 are required for RNA m^6^A methylation, the phenotypic differences of the two cell lines indicate there might be methylation-independent effects. To evaluate their roles in RNA methylation, we therefore isolated nonribosomal RNAs from soluble cytoplasmic, nucleoplasmic, and chromatin-associated fractions and quantified m^6^A levels with liquid chromatography–tandem mass spectrometry (LC–MS/MS). The m^6^A/A ratios in all three fractions showed a comparable decrease upon either *Mettl3* or *Mettl14* knockout (Fig. 1C and 1D). To further specify the methylation substrates of METTL3 and METTL14, we immunoprecipitated m^6^A-containing chromatin-associated RNAs (caRNAs, ribosomal-RNA depleted) or mRNAs and performed high-throughput sequencing (m^6^A-MeRIP-seq) in the two CKO cell lines and their respective controls. The m^6^A level on mRNAs and caRNAs reduced similarly in both CKO cells (Fig. S2A and S2B), consistent with the LC-MS/MS analysis (Fig. 1C and 1D). We also detected a low level of *N*^*6*^, *N*^*6*^-dimethyladenosine modification (m^6^_2_A) from LC–MS/MS in our samples, the RNA modification only present on rRNAs (Sergiev et al., 2018), and lower than 1% of rRNA reads from m^6^A-MeRIP-seq (Fig. S2C and S2D), suggesting limited rRNA contamination.

In each cell line, we identified ~30,000 peaks in the caRNAs and ~20,000 peaks in the mRNAs that are highly reproducible between two biological replicates (Fig. S2E and S2F). Compared to the controls, *Mettl3* and *Mettl14* knockout cells both showed more hypo-methylated peaks on mRNAs and caRNAs (Fig. S2G and S2H). Our previous work has demonstrated that METTL3 can deposit m^6^A on carRNAs, and thus tune chromatin state and transcription (Liu et al., 2020). We then examined the m^6^A methylation on carRNAs, namely promoter-associated RNA, enhancer RNAs, and repeats RNA. As expected, the *Mettl14* knockout resulted in an overall reduction in the carRNA methylation levels (Fig. 1E and 1F). We noticed that in all carRNA species, the m^6^A fold-changes upon *Mettl3* and *Mettl14* knockout are positively correlated (Fig. S2I), suggesting that METTL14 can regulate carRNAs methylation in a manner similar to, although not identical to METTL3. Consistent with our findings (Liu et al., 2020), such decrease in m^6^A levels on carRNAs did lead to an increase in RNA abundance upon either *Mettl3* or *Mettl14* knockout in mESCs (Fig. 1G and 1H). We also performed functional enrichment analysis with genes with hypo-methylated RNAs upon *Mettl14* or *Mettl3* knockout and found that METTL3 and METTL14 were involved in similar pathways through m^6^A methylation (Fig. S3A and S3B). Overall, we barely detected difference in the effects of METTL3 and METTL14 on m^6^A methylation in mESCs, supporting that both proteins are required for methylation.

**Figure 2.**
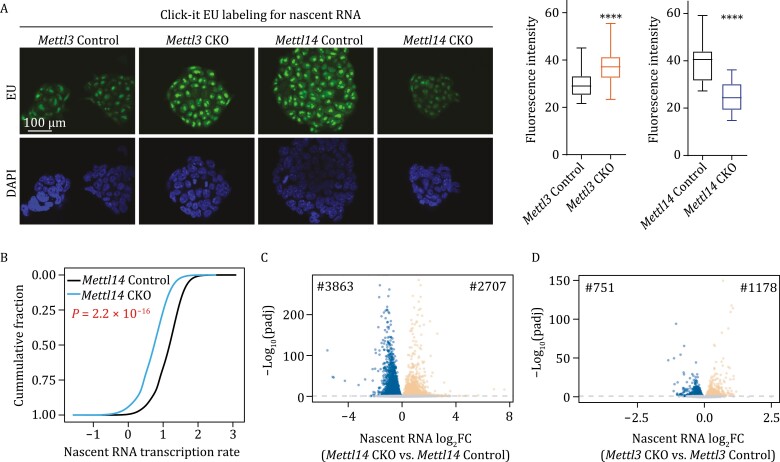
**METTL3 and METTL14 showed opposite effects on transcription regulation.** (A) Nascent RNA synthesis in *Mettl3* Control, *Mettl3* CKO, *Mettl14* Control, and *Mettl14* CKO mESCs, detected by using a click-it RNA Alexa fluor 488 imaging kit. EU, 5-ethynyl uridine; DAPI, 4ʹ,6-diamidino-2-phenylindole. (B) Cumulative distributions of transcription rate in *Mettl14* Control and *Mettl14* CKO in mESCs. *P* values were calculated by a nonparametric Wilcoxon-Mann–Whitney test. (C and D) Volcano plots of genes that differentially expressed upon *Mettl14* (C) or *Mettl3* (D) knockout in mESCs (adjusted *P* [padj] < 0.05). Down- and up-regulated genes are highlighted with blue and orange, respectively.

To further explore why the two knockout cell lines behave differently, we quantified the transcription rate of nascent RNAs. Depletion of METTL3 is known to induce more open chromatin and elevate transcription in mESCs (Liu et al., 2020). Surprisingly, nascent transcript synthesis was inhibited in *Mettl14* knockout mESCs compared to the control, contrary to *Mettl3* knockout cells (Figs. 2A, 2B, and S3C). Consistent with lower transcription rate, *Mettl14* knockout also induced more nuclear RNA downregulation (3,863 down-regulated genes vs. 2,707 up-regulated genes), while *Mettl3* knockout caused more nuclear RNA upregulation (751 down-regulated genes vs. 1,178 up-regulated genes) (Fig. 2C and 2D). Furthermore, the genes that were downregulated by *Mettl14* knockout and also upregulated by *Mettl3* knockout are enriched with signaling pathways regulating pluripotency of stem cells (Fig. S3D and S3E). Considering that METTL3 and METTL14 barely differed in m^6^A methylation deposition, but showed distinct effects on phenotypes and transcriptome responses, we suspected that METTL14 is involved in chromatin regulation independent of m^6^A methylation.

### METTL14 shows distinct chromatin-binding preference from that of METTL3

**Figure 3.**
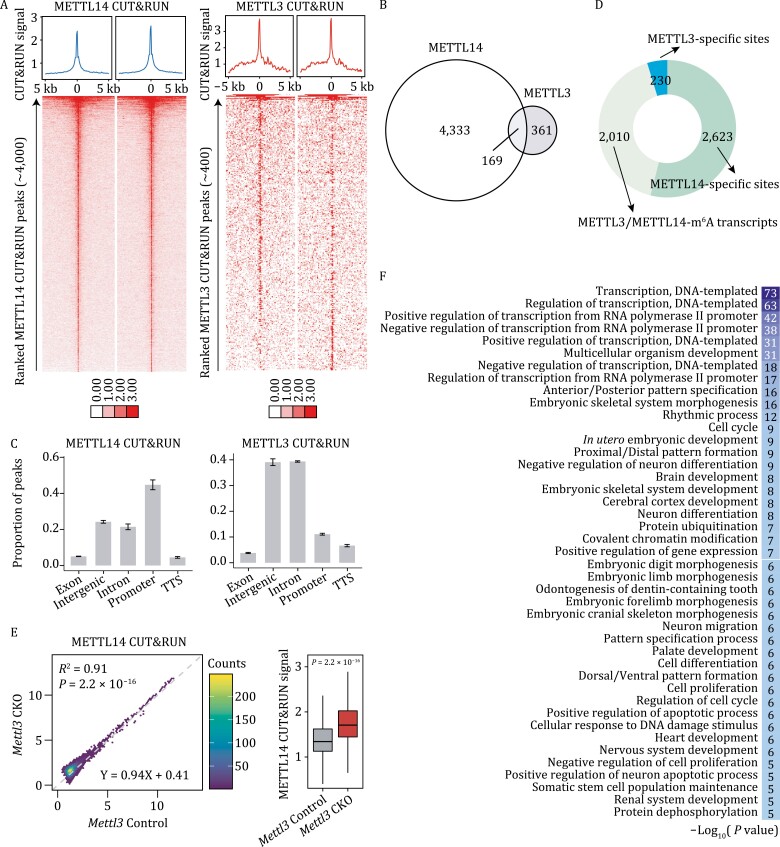
**METTL14 displayed distinct chromatin bindings compared with METTL3.** (A) Heatmap showing METTL14 CUT&RUN signal around the METTL14 peak centers (±5 kb, *left* panel), and METTL3 CUT&RUN signal around the METTL3 peak centers (±5 kb, *right* panel) in wild-type mESCs. (B) Venn diagram of peaks overlap between METTL3 and METTL14 CUT&RUN peaks in wild-type mESCs. (C) Distribution of METTL3 and METTL14 CUT&RUN peaks in wild-type mESCs at distinct genomic regions including promoter, TTS, exonic, intronic, and intergenic regions annotated by HOMER in *Mettl3* Control and *Mettl3* CKO mESCs. *n* = 2 biological replicates; error bars indicate means ± SEM. (D) METTL14 and METTL3 sites in wild-type mESCs were categorized into three groups: (i) METTL14 binding sites that were neither colocalized with METTL3 nor located at m^6^A-marked transcripts (METTL14-specific sites); (ii) METTL3 bindings sites that were neither colocalized with METTL14 nor located at m^6^A-marked transcripts (METTL3-specific sites); (iii) METTL14 or METTL3 binding sites that were located at m^6^A-marked transcripts (METTL3/METTL14-m^6^A transcripts). Bar chart shows the number of sites in each group. The caRNA MeRIP-seq in this study were used to identify m^6^A-marked transcripts. (E) Scatter plot (left panel) and boxplot (right panel) showing METTL14 CUT&RUN signal in *Mettl3* Control and *Mettl3* CKO mESCs. *P* value in boxplot was calculated by a nonparametric Wilcoxon-Mann–Whitney test. (F) Gene Ontology (GO) enrichment analysis of METTL14 gene targets in mESCs.

To investigate the role of METTL14 on chromatin, we performed CUT&RUN to map the genomic binding of METTL14 and METTL3 in mESCs. In wild-type mESCs, we captured ~400 genomic binding sites for METTL3, and more than 4,000 genomic binding sites for METTL14 (Fig. 3A), with a highly specific and well-validated antibody (Fig. S4A and S4B). This observation led us to hypothesize that METTL14, with intense genomic binding sites, may play a different chromatin regulatory role compared to METTL3.

We overlapped the binding sites of METTL14 with METTL3 in wild-type mESCs, with only ~4% of the genomic binding sites of METTL14 colocalized with those of METTL3 (Fig. 3B). We then categorized METTL14 and METTL3 peaks according to their locations on functional elements and found that METTL14 favored promoter regions, while METTL3 preferred intergenic and intron regions (Fig. 3C). Next, to investigate how METTL14 differed from METTL3 in chromatin binding, we overlaid their respective genomic binding sites with various euchromatin and heterochromatin histone marks (Fig. S4C and S4D). We found that METTL3 loci are predominantly enriched with the repressive histone mark H3K9me3, which is consistent with a recent finding that METTL3 is recruited to H3K9me3 decorated genomic loci (mainly localized at the intergenic regions) by an H3K9me3 methyltransferase SETDB1 in mESCs (Xu et al., 2021). However, METTL14 binding sites are associated with repressive histone mark H3K27me3 rather than H3K9me3, and also with active histone marks H3K27ac and H3K4me3 (Fig. S4C and S4D), further supporting that METTL14 has different chromatin-binding preferences from METTL3.

We next asked whether the difference in genomic binding of METTL14 and METTL3 is associated with RNA m^6^A methylation. We first categorized METTL14 and METTL3 binding sites in wild-type mESCs into three groups based on their relationship with m^6^A: (i) METTL14 binding sites that are neither colocalized with METTL3 nor located at m^6^A-marked transcripts (METTL14-specific sites); (ii) METTL3 bindings sites that are neither colocalized with METTL14 nor located at m^6^A-marked transcripts (METTL3-specific sites); (iii) METTL14 or METTL3 binding sites that are located at m^6^A-marked transcripts (METTL3/METTL14-m^6^A transcripts) (Fig. 3D). Apart from ~2,000 METTL3/METTL14-m^6^A transcripts and 230 METTL3-specific sites, we identified 2,623 METTL14-specific genomic binding sites, accounting for ~60% of its total bindings. We further overlapped the three groups of binding sites with various histone marks and observed that METTL3/METTL14-m^6^A sites are mainly located at genomic regions with active histone marks H3K4me3 and H3K27ac, consistent with m^6^A being installed at actively transcribed regions by m^6^A MTCs (Fig. S4E). In addition to the two active histone marks H3K4me3 and H3K27ac, METTL14-specific sites overlap mostly with repressive marks, particularly H3K27me3, while METTL3-specific sites overlap mostly with H3K9me3 (Fig. S4D and S4E). These results suggested that METTL14 and METTL3 could play diverse roles: on one hand, they form heterodimer to co-transcriptionally install m^6^A at actively transcribed regions, on the other hand, they bind chromatin with distinct preference and may function independently of their roles in m^6^A installation.

We then examined whether the chromatin binding of METTL14 and METTL3 are affected by each other. We mapped the genomic binding of METTL14 in both control and *Mettl3* knockout mESCs as well as METTL3 binding sites in the control and *Mettl14* knockout mESCs (Fig. S4F and S4G). The genomic binding of METTL14 globally increased upon *Mettl3* depletion (Fig. 3E). In contrast, the global binding of METTL3 in *Mettl14* knockout mESCs showed a general reduction compared to the control (Fig. S4H), suggesting that METTL3 binding on chromatin is dependent on METTL14. Since we already found that *Mettl14* knockout repressed transcription globally (Fig. 2A–C), we then asked if this transcriptional repression is associated with the different chromatin-binding preference of METTL14 and METTL3. We profiled their genomic binding on genes that were significantly repressed after *Mettl14* knockout (3,863 down-regulated genes in Fig. 2C). Although these down-regulated genes were occupied by both METTL14 and METTL3, no obvious changes in METTL3 binding were observed upon *Mettl14* knockout, suggesting that METTL3 plays limited roles in these down-regulated genes (Fig. S4I). In this way, it is the chromatin binding of METTL14, but not METTL3, that is critical to genes repression upon *Mettl14* knockout. In addition, the GO enrichment analysis of the METTL14-targeted genes showed that they were mainly involved in transcriptional regulation and embryonic development (Fig. 3F). Taken together, these results suggested that the chromatin binding of METTL14 but not m^6^A methylation could be responsible for the transcription regulation and morphologies caused by depletion of *Mettl14*, which is distinct from *Mettl3* knockout in mESCs.

### METTL14 regulates H3K27me3 deposition at their co-occupied facultative heterochromatin

**Figure 4.**
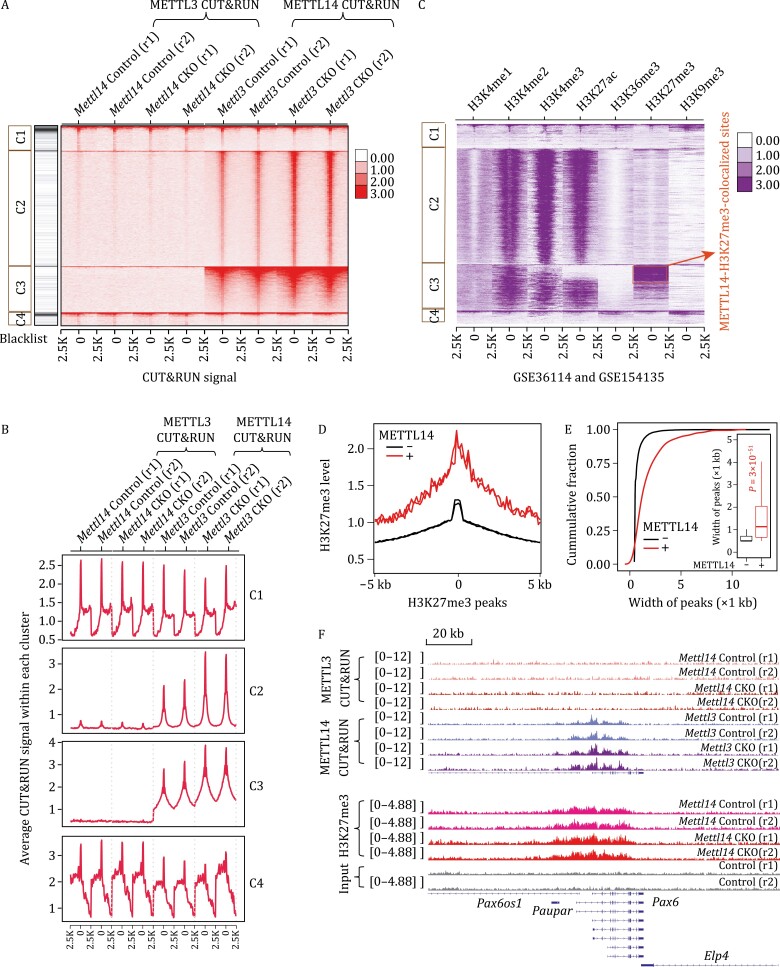
**METTL14 colocalizes with H3K27me3 marked facultative heterochromatin.** (A) METTL3 and METTL14 CUT&RUN peaks are grouped into four clusters with K-means clustering method. The inputs are METTL3 or METTL14 CUT&RUN signals at their peak centers and the flanking 2.5 kb regions in *Mettl3* Control and *Mettl3* CKO, *Mettl14* Control and *Mettl14* CKO mESCs. The heatmap showing the clustering results of METTL3 and METTL14 CUT&RUN signal on their peak centers and the flanking 2.5 kb regions. (B) Average profiles of METTL3 and METTL14 CUT&RUN signal within each cluster in (A). (C) The heatmap showing various histone modification on the four clusters of METTL3 and METTL14 CUT&RUN peak centers and the flanking 2.5 kb regions identified in (A). (D) Average profile of H3K27me3 modification level around H3K27me3 peak center and the flanking 5 kb regions. H3K27me3 peaks were categorized into METTL14 bound (+) and unbound (−) groups. (E) Cumulative distribution and boxplots (inside) of H3K27me3 peaks width. H3K27me3 peaks were categorized into METTL14 bound (+) and unbound (−) groups. *P* values were calculated by a nonparametric Wilcoxon-Mann–Whitney test. (F) IGV plots showing METTL3 and METTL14 CUT&RUN signal, H3K27me3 modification level and its inputs around *Pax6* gene loci in *Mettl14* Control and *Mettl14* CKO mESCs, respectively.

To unbiasedly compare genomic bindings between METTL14 and METTL3, we applied the unsupervised K-means clustering method. We used the binding intensity of METTL14 and METTL3 around their peak centers (±2.5 kb) in *Mettl3* or *Mettl14* knockout mESCs and their respective controls as input, and classified METTL3 and METTL14 genomic bindings into four clusters (C1–4) (Fig. 4A). Among the four clusters, C1 and C4 showed significant METTL3 binding but they differed in METTL14 binding intensity. METTL14 majorly binds to C2 and C3, with the latter showing a much higher METTL14 binding intensity and being completely devoid of METTL3 binding (Fig. 4B). To further characterize chromatin binding of METTL14 and METTL3 in the four clusters, we overlaid them with various euchromatin and heterochromatin histone marks. As we expected, METTL3-bound clusters C1 and C4 enriched H3K9me3, similar to METTL3-specific sites; METTL14-bound cluster C2 was predominantly modified by H3K4me3 and H3K27ac, more like the METTL3/METTL14-m^6^A sites; and METTL14-bound cluster C3 enriched both active histone marks H3K4me3 and H3K27ac and repressive histone mark H3K27me3, corresponding to the METTL14-specific sites (Fig. 4C). Intriguingly, distinct chromatin states could be further identified through categorizing binding intensities of METTL14 in the C3 cluster. The moderate bindings of METTL14 denoted active histone modification H3K4me3 and H3K27ac, whereas the strong bindings of METTL14 were colocalized with repressive histone mark H3K27me3 (hereafter referred to as the METTL14-H3K27me3-colocalized sites) (Figs. 4C and S5A), suggesting that chromatin-binding strength of METTL14 is closely associated with different chromatin states. Likewise, H3K27me3 at regions occupied by METTL14 displayed a higher modification level with broader peaks compared to those without METTL14 binding (Figs. 4D–4F, S5B and S5C). These METTL14-H3K27me3-colocalized sites showed lower transcription levels compared to the ones bound by METTL14 but not marked with H3K27me3, which is consistent to the repressive roles of H3K27me3 (Fig. S5D). In addition, these METTL14-H3K27me3-colocalized sites were also devoid of m^6^A methylation (Fig. S5E), consistent with the observation that these H3K27me3-marked and METTL14-bound loci are devoid of METTL3 binding and thus no m^6^A deposition. Altogether, these METTL14-H3K27me3-colocalized loci are associated with the function of METTL14 as a chromatin regulator that is independent of METTL3 and m^6^A.

**Figure 5.**
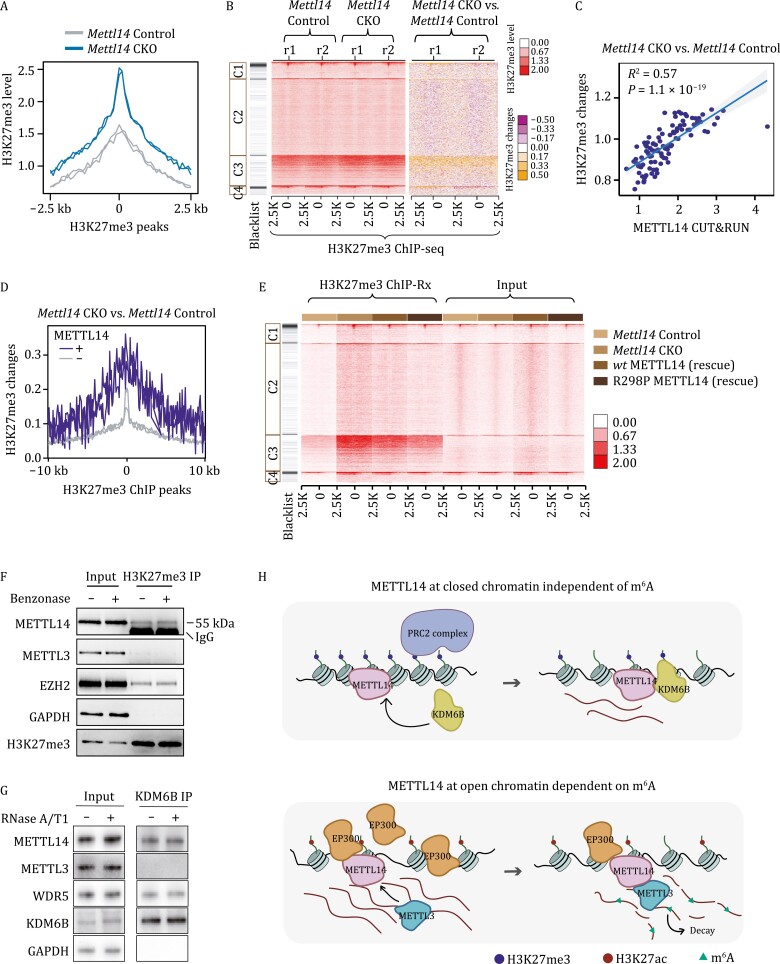
**METTL14 directly regulates H3K27me3 deposition in mESCs.** (A) Average profiles of H3K27me3 modification at H3K27me3 peak centers and the flanking 2.5 kb regions in *Mettl14* Control and *Mettl14* CKO mESCs, respectively. *n* = 2 biological replicates. (B) Heatmap showing H3K27me3 level (left panel) and H3K27me3 level changes (right panel) upon *Mettl14* knockout in mESCs, respectively. (C) The correlation between METTL14 CUT&RUN signal and H3K27me3 changes upon *Mettl14* knockout in mESCs, respectively. Sites ranked by METTL14 CUT&RUN signal were grouped and average into 100 data point. (D) Average profile of H3K27me3 changes upon *Mettl14* knockout in mESCs at H3K27me3 peak centers and the flanking 10kb regions. H3K27me3 peaks were categorized into METTL14 bound (+) and unbound (−) groups. (E) Heatmap showing H3K27me3 levels at four identified clusters in *Mettl14* Control, *Mettl14* CKO, and *Mettl14* CKO mESCs rescued with wild-type METTL14 or R298P mutated METTL14. ChIP-seq signal has been normalized to *Drosophila* Spike-in DNAs. (F) Western blots of the immunoprecipitated H3K27me3 and its interaction with METTL14 in mESCs with and without Benzonase treatment. (G) Western blots of the immunoprecipitated KDM6B and its interaction with METTL14 and METTL3 in mESCs with and without RNase treatment. (H) A schematic model showing how METTL14 functions distinctly on chromatin when located at repressive (upper panel) and active (lower panel) chromatin, respectively.

To assess the regulatory effects of METTL14 on the H3K27me3 modification, we proceeded to perform Chromatin Immunoprecipitation Sequencing (ChIP-seq) of H3K27me3 in control and *Mettl14* knockout mESCs. In addition, *Mettl14* knockout led to an overall increase in repressive histone mark H3K27me3 (Figs. 5A and S6A), which was also validated with both Western blot and CUT&RUN (Fig. S6B–D). To further correlate changes of H3K27me3 in *Mettl14* knockout mESCs with chromatin binding of METTL14, we profiled changes in H3K27me3 in our four clusters (C1–C4). The increases in H3K27me3 after *Mettl14* knockout occurred mainly in the METTL14-H3K27me3-colocalized C3 cluster (Fig. 5B). Additionally, the increase of H3K27me3 upon *Mettl14* depletion was significantly correlated with the binding density of METTL14 in wild-type mESCs (Fig. 5C). And these changes were observed only on the METTL14 genomic loci rather than those also with the presence of METTL3 (Fig. S6E). Furthermore, upon *Mettl14* knockout in mESCs, the METTL14-H3K27me3-colocalized loci showed a greater increase in H3K27me3 modification, compared to those not bound by METTL14 (Fig. 5D), along with a more decreased RNA expression level (Fig. S6F). These results further confirmed that METTL14, but not METTL3, hinders the accumulation of H3K27me3 through its direct chromatin binding at the METTL14-H3K27me3-colocalized loci.

To further decipher the regulatory roles of METTL14 on H3K27me3 and its dependence on m^6^A, we generated stable rescue cell lines that express wild-type METTL14 or an inactive R298P mutant (methyltransferase activity abrogated) (Sledz and Jinek, 2016; Wang et al., 2016a, 2016b; Liu et al., 2018). We proceeded with ChIP-seq of H3K27me3 in *Mettl14* control, *Mettl14* CKO together with the two rescued *Mettl14* CKO cell lines. The increased level of H3K27me3 observed in *Mettl14* CKO mESCs was reversed with both wild-type and inactive mutant METTL14, which was consistent with Western blot results (Figs. 5E and S6G–I). These results all support that the function of METTL14 as a chromatin regulator of H3K27me3 is independent of its function as a component of methyltransferase.

To further explore the mechanism of how METTL14 regulate H3K27me3, we did co-immunoprecipitation (co-IP) experiments in wild-type mESCs with and without Benzonase (an endonuclease degrades all forms of DNA and RNA) treatment to test whether METTL14 could bind H3K27me3. We found that METTL14, but not METTL3, directly binds H3K27me3 independent of both RNA and DNA (Fig. 5F), in consistent with our results that METTL14 can colocalize with H3K27me3 (Fig. 4C). We further confirmed the direct interaction of METTL14 with H3K27me3 by performing an *in vitro* co-IP experiment with the H3K27me3-containing peptide (Fig. S6J). A recent study reported that METTL14 can interact with lysine demethylase 6B (KDM6B) to facilitate demethylation of the adjacent H3K27me3 (Wu et al., 2020). We performed co-IP to confirm METTL14, but not METTL3, interacts with KDM6B (Figs. 5G and S6K). The interaction between KDM6B and METTL14 is independent of RNA, and not dependent on other components of the MTC (Fig. S6L). Besides, when treated mESCs with GSK-J4, a KDM6B inhibitor, we observed an overall decrease in nascent RNA synthesis as expected (Fig. S6M). Taken together, we propose that METTL14 depletion disrupts the interaction between METTL14 and KDM6B in the METTL14-H3K27me3-colocalized genomic regions, which leads to an increased H3K27me3 level and downstream gene repression, indicating that METTL14 depletion impairs the recruitment of KDM6B, leading to the accumulation of H3K27me3 (Fig. 5H).

### METTL14 regulates H3K27me3 changes during neuronal differentiation of mESCs

**Figure 6.**
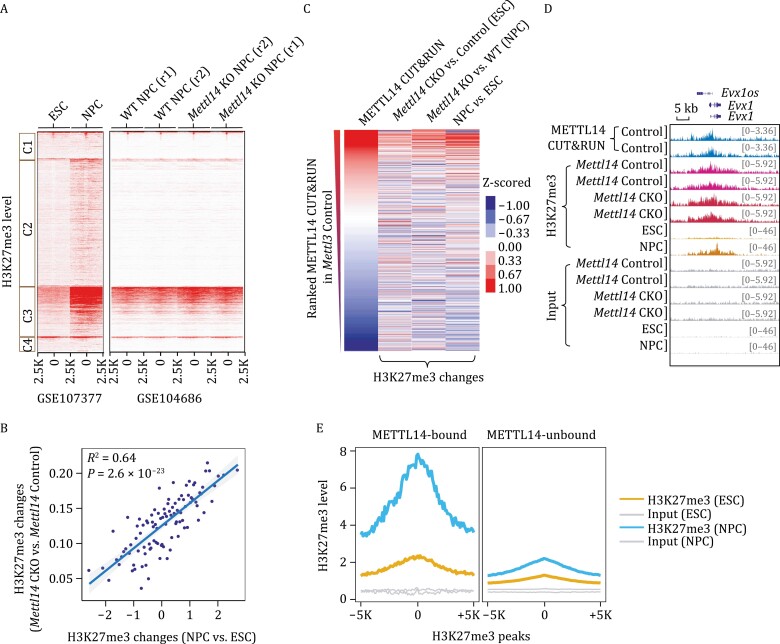
**METTL14 regulates H3K27me3 changes during neuronal differentiation of mESCs.** (A) Heatmap showing H3K27me3 levels on four clusters of METTL3 and METTL14 CUT&RUN peak centers and the flanking 2.5 kb regions in ESCs, NPCs, wildtype (WT), and *Mettl14* KO NPCs, respectively. (B) The correlation of H3K27me3 changes between NPCs vs. ESCs and *Mettl14* CKO vs. *Mettl14* Control ESCs. Sites ranked by H3K27me3 changes upon differentiation (NPC vs. ESCs) were grouped and average into 100 data point. (C) Heatmap showing METTL14 CUT&RUN signal, and changes of H3K27me3 level comparing *Mettl14* CKO vs. *Mettl14* Control ESCs, *Mettl14* KO vs. wildtype (WT) NPC, NPC vs. ESCs, respectively on METTL14 CUT&RUN peak centers and the flanking 2.5 kb regions. METTL14 peaks were ranked by its CUT&RUN signal intensity. (D) IGV plots of METTL14 CUT&RUN signal and H3K27me3 modification in *Mettl14* Control and *Mettl14* CKO mESCs, wild-type ESCs and NPCs around *Evx1* gene loci. (E) Average profile of H3K27me3 in mESCs and NPCs at H3K27me3 peak centers and the flanking 5KB regions. H3K27me3 peaks were categorized into METTL14 bound (METTL14-bound) and unbound (METTL14-unbound) groups.

H3K27me3 is critical for mammalian development (Wiles and Selker, 2017), and we did notice a dramatic increase in H3K27me3 levels at the METTL14-H3K27me3-colocalized genomic regions during mESC differentiation (Fig. S7A), in particular differentiation into neurons (Fig. 6A). A recent study showed that *Mettl14* knockout led to premature differentiation of neural stem cells (Wang et al., 2018). We then asked whether the H3K27me3 modification at METTL14-H3K27me3-colocalized genomic regions would regulate neuronal differentiation. We ranked genomic H3K27me3 peaks according to their level changes during the differentiation of mESCs into neural progenitor cells (NPCs). We found that the increase of H3K27me3 during differentiation was positively correlated with H3K27me3 changes upon *Mettl14* knockout (R^2^ = 0.64; Fig. 6B), suggesting a regulatory role of METTL14 on H3K27me3 during neuronal differentiation.

Next, to examine whether METTL14 chromatin bindings are involved in H3K27me3 regulation during neuronal differentiation, we ranked METTL14-bound genomic loci based on their binding intensity in wild-type mESCs and found that the higher the binding intensity of METTL14 the greater the increase of H3K27me3 during differentiation through neuronal lineage (R^2^ = 0.64; Figs. 6C and S7B). Similar trends were also observed upon *Mettl14* depletion in mouse NPCs (R^2^ = 0.55) (Figs. 6C and S7C). Furthermore, when categorizing the H3K27me3 peaks into METTL14-bound and METTL14-unbound groups, we found that the increase in H3K27me3 during neuronal differentiation predominantly occurred at the METTL14-H3K27me3-colocalized loci (Fig. 6D and 6E), suggesting that METTL14 chromatin binding is essential to the differentiation of mESCs into NPCs and this process hinges upon H3K27me3 regulation.

### H3K27me3 methyltransferase inhibitor can rescue the effects of *Mettl14* knockout in embryoid body formation of mESCs

**Figure 7.**
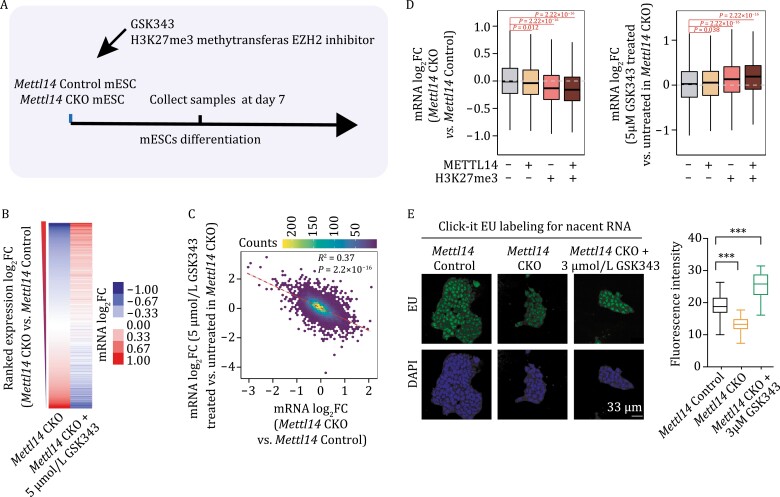
**H3K27me3 methyltransferase inhibitor rescued gene expression changes upon *Mettl14* depletion.** (A) Schematic of experimental design. (B) Heatmap showing gene expression fold-changes (log_2_FC) comparing *Mettl14* CKO vs. *Mettl14* Control mEBs, *Mettl14* CKO mEBs treated with GSK343 vs. untreated, respectively. Genes were ranked by gene expression log_2_FC comparing *Mettl14* CKO vs. *Mettl14* Control mEBs. (C) The correlation of mRNA expression log_2_FC between *Mettl14* CKO vs. *Mettl14* Control, and *Mettl14* CKO mEBs treated with GSK343 vs. untreated. (D) Boxplots of mRNA expression log_2_FC comparing *Mettl14* CKO vs. *Mettl14* Control mEBs (left panel), and *Mettl14* CKO mEBs treated with GSK343 vs. untreated (right panel). Genes were categorized into four groups according to whether they are targets of H3K27me3 or METTL14. *P* values were calculated by a nonparametric Wilcoxon-Mann–Whitney test. (E) Nascent RNA synthesis in *Mettl14* Control, *Mettl14* CKO and *Mettl14* CKO treated with 3 μmol/L GSK343 mESCs, detected by using a click-it RNA Alexa fluor 488 imaging kit. EU, 5-ethynyl uridine; DAPI, 4ʹ,6-diamidino-2-phenylindole.

To determine whether the increased level of H3K27me3 is responsible for the altered pluripotency states of mESCs upon *Mettl14* knockout, we sought to rescue the phenotype with chemical inhibitors that can abrogate the activities of H3K27me3-related enzymes. The commercially available inhibitor, GSK343 (against H3K27me3 methyltransferase EZH2) was selected. As knockout of *Mettl14* caused a global increase in H3K27me3 levels (Fig. 5A), we treated *Mettl14* knockout mESCs with GSK343. We collected cells after they differentiated into embryonic bodies (EBs), extracted RNAs, and performed RNA-seq to evaluate RNA expressions (Fig. 7A). As expected, the expression changes induced by *Mettl14* knockout were largely rescued when treated with GSK343 (Fig. 7B and 7C). To further assess the impacts of the chromatin binding of METTL14, we categorized genes into four groups according to whether they were bound by METTL14 or marked with H3K27me3, respectively. We observed that when we treated *Mettl14* knockout mEBs with GSK343, the expression changes of genes were maximally rescued at the METTL14-H3K27me3-colocalized regions (Fig. 7D). To investigate if the m^6^A methylation might also affect global transcriptome upon *Mettl14* knockout in mEBs, we categorized genes into m^6^A-marked and H3K27me3-modified subgroups. Though m^6^A-marked genes also showed a global reduction in gene expression upon *Mettl14* knockout, the extent of this reduction cannot be compared with expression changes of H3K27me3-modified genes (Fig. S7D), suggesting that the methylation-independent, chromatin regulation role of METTL14 can be prominent. Furthermore, treatment with GSK343 partially rescued the reduced nascent RNA synthesis, as well as the disrupted colony morphology caused by *Mettl14* depletion in mESCs (Figs. 7E and S7E). Taken together, our results demonstrated that the regulation of H3K27me3 by METTL14, especially via its direct chromatin bindings, is critical to the pluripotency maintenance and differentiation of mESCs.

## Discussion

To date, the function of m^6^A in mammalian development is still mysterious. We revealed that METTL14 has an additional function independent of m^6^A methylation, it regulates mammalian heterochromatin in a manner distinct from METTL3. This m^6^A-independent function is critical to the differential phenotype and transcriptional regulation in mESCs.

We demonstrated that depletion of *Mettl14* induced an overall increase in H3K27me3, especially at regions co-occupied by METTL14 and H3K27me3. Our results support that METTL14 antagonizes the accumulation of H3K27me3 in mESCs: (i) METTL14 rather than METTL3 can bind H3K27me3 in an RNA- and m^6^A-independent manner; (ii) METTL14 interacts with H3K27me3 demethylase KDM6B; (iii) H3K27me3 level at METTL14-H3K27me3-occupied genomic loci increased after *Mettl14* knockout. Taken together, METTL14 impedes the accumulation of H3K27me3 at these regions through its direct interaction with the H3K27me3 demethylase KDM6B. Furthermore, treatment with GSK343, an H3K27me3 methyltransferase inhibitor, rescued changes in gene expression upon *Mettl14* depletion, especially for genes co-occupied by METTL14 and H3K27me3, demonstrating that the regulation of METTL14 on H3K27me3 at the METTL14-H3K27me3-co-occupied genomic regions are critical to the self-renewal and differentiation ability of mESCs.

In addition to H3K27me3, METTL14 genomic binding can also colocalize with activate histone modifications H3K4me3 and H3K27ac, and METTL14 interacts with components of active gene regulation complexes. These active regions are enriched with m^6^A-modified transcripts, suggesting the presence of binding partners of METTL14 on active chromatin that may help recruit the heterodimer of METTL14 and METTL3 to install m^6^A. However, transcripts derived from the genomic regions co-occupied by METTL14 and H3K27me3 are less enriched of m^6^A, suggesting that alternative METTL14 binding partners on these regions prevent the binding of METTL3. Therefore, our results suggest that the binding partners of METTL14 at active and repressive genomic regions recognize different domain/pocket of METTL14 to dictate diverse or even opposite functions (Fig. 5H). Recent work also suggested that METTL3/METTL14 has methylation-independent functions (Lin et al., 2016; Liu et al., 2021b). We show here METTL14 can recruit KDM6B for H3K27me3 demethylation and is itself a chromatin regulator. This methylation-independent function is critical to early development. It is also important to carefully analyze the m^6^A-dependent and m^6^A-independent functions and pathways in different biological systems when modulating METTL14 levels. In many cases, the m^6^A-dependent pathways may dominate through recruiting both METTL3 and METTL14, in other cases the m^6^A-independent pathway may dominate through recruiting just METTL14 as shown in the mESC differentiation into neuronal lineage in this study. Future research should also address how METTL14 alone is recruited without METTL3 and its importance in other biological processes.

## Materials and methods

### Mouse embryonic stem cells construction

The CKO *Mettl14* C57BL/6 mice were a generous gift from Dr. Ming-Han Tong’s group (Lin et al., 2017), CKO *Mettl14* ESCs were derived from *Mettl14*^*flox*/*flox*^ blastocyst. 2 × 10^5^ mESC cells were then transfected with 200 ng PB-CAG-Puromycin-P2A-CreERT2 and 100 ng PBase by electroporation. After 24 h, electroporated cells were treated with 1 μg/mL Puromycin to generate stable *Mettl14*^*flox*/*flox*^; *CreERT2* ES clones (CKO *Mettl14*). *Mettl14* CKO cells were electroporated with PB-CAG-m*Mettl14*-P2A-blasticidin or PB-CAG-m*Mettl14*-R298P-P2A-blasticidin, 24 h after electroporation, 1 μg/mL Puromycin and 10 μg/mL blasticidin were added to generate stable rescued wild-type (*wt*) or R298P mutated (*mu*) *Mettl14* mESCs.

CKO *Mettl3* C57BL/6 ESCs were generated by inserting a FLIP cassette into the exon 3 of *Mettl3* assisted by CRISPR/Cas9 as described before (Andersson-Rolf et al., 2017). Once the Cre recombinase is activated, the FLIP cassette will be inverted to a mutagenic configuration to disrupt the splicing of *Mettl3* gene, resulting in the complete loss of gene function. 2 × 10^5^ mESC cells were then transfected with 200 ng PB-CAG-Puromycin-P2A-CreERT2 and 100 ng PBase by electroporation. After 24 h, electroporated cells were treated with 1 μg/mL Puromycin to generate stable *Mettl3*^*flox*/*flox*^; *CreERT2* ES clones (CKO *Mettl3*).

### Mouse embryonic stem cells culture

We cultured mESCs in DMEM (Invitrogen) supplemented with 15% FBS (GeminiBio), 1% nucleosides (100×) (Millipore), 1 mmol/L l-glutamine (Gibco), 1% nonessential amino acid (Gibco), 0.1 mmol/L 2-mercaptoethanol (Sigma), 1,000 U/mL LIF (Millipore), 3 μmol/L CHIR99021 (Stemcell), and 1 μmol/L PD0325901 (Stemcell) in 37°C and 5% CO_2_.

### Nascent RNA labeling assay

mESCs cells were grown on pre-coated glass over slides. After 24 h, the nascent RNA synthesis assay was performed by using the Click-It RNA Imaging Kits following the manufacturer’s protocol. Olympus FV1000 was used for confocal image acquisition, and Image J software (Schneider et al., 2012) was used to quantify the signal intensity.

### Western blot

The samples were treated with RIPA buffer containing 1× protease inhibitor cocktail. The cell lysates were then mixed with 4× loading buffer and boiled at 98°C for 15 min and stored at −80°C for use in the next step. A total of 30 μg protein of each sample was loaded into 4%–12% NuPAGE Bis-Tris gel and then transferred to PVDF membrane. The membranes were blocked in 5% milk PBST for 1 h at room temperature (RT), and incubated in a diluted primary antibody solution in 5% milk PBST at 4°C overnight. The membranes were then washed and incubated in a dilution of secondary antibody conjugated to HRP for 1 h at RT. Protein bands were detected using Pierce™ ECL Western Blotting Substrate kit.

### Cell fractionation

mESCs were fractionated following the procedure previously published (Wuarin and Schibler, 1994). Briefly, 5 × 10^6^ to 1 × 10^7^ cells were collected, washed with 1 mL cold PBS/1 mmol/L EDTA buffer, and collected by centrifugation at 500 ×*g*. Added 200 μL ice-cold lysis buffer (10 mmol/L Tris-HCl, pH = 7.5, 0.05% NP40, 150 mmol/L NaCl) to the cell pellet and incubated on ice for 5 min, then gently pipetted up the cell lysate over 2.5 volumes of chilled sucrose cushion (24% RNase-free sucrose in lysis buffer), and the cell pellet was collected by centrifuging with 15,000 ×*g* for 10 min at 4°C. All the supernatant was collected as cytoplasmic fraction. Then gently added 200 μL ice-cold PBS/1mmol/L EDTA to the nuclei pellet without disturbing the pellet, then aspirated the PBS/EDTA. Added 100 μL prechilled glycerol buffer (20 mmol/L Tris-HCl, pH = 7.9, 75 mmol/L NaCl, 0.5 mmol/L EDTA, 0.85 mmol/L DTT, 0.125 mmol/L PMSF, 50% glycerol) to resuspend the nuclei pellet with gentle flicking of the tube, then added an equal volume of cold nuclei lysis buffer (10 mmol/L HEPES, pH = 7.6, 1 mmol/L DTT, 7.5 mmol/L MgCl_2_, 0.2 mmol/L EDTA, 0.3 mol/L NaCl, 1 mol/L UREA, 1% NP40) then vigorously vortexed for 1 min. The mixtures of nuclei pellet were incubated on ice for 5 min, then centrifuged at 4°C with 15,000 ×*g* for 2 min. Collected all the supernatant as soluble nuclear fraction/nucleoplasm, then gently rinsed the pellet with cold PBS/1 mmol/L EDTA then collected as chromosome-associated fraction.

### RNA isolation

TRIZOL was used to isolate total RNA following the manufacturer’s instructions. RiboMinus transcriptome isolation kit was used to extract nonribosomal RNA from the total RNA. Dynabeads mRNA DIRECT™ kit was used to extract mRNA from total RNA. The RNA concentration was measured by UV absorbance at 260 nm.

### LC-MS/MS quantification of m^6^A in nonribosomal RNA

A hundred nanograms of nonribosomal RNA/mRNA was digested with nuclease P1 (1 U) in 25 μL of buffer containing 20 mmol/L NH4Ac at 42°C for 2 h, then NH4HCO3 (1 mol/L, 3 μL) and alkaline phosphatase (0.5 U) were added and incubated at 37°C for 2 h. After the digestion steps, sample were filtered (0.22 µm pore size, 4 mm diameter), and 5 μL of the diluted sample (1:1 diluted) was injected into the LC-MS/MS. The nucleosides were separated by reverse phase ultra-performance liquid chromatography on a C18 column, and detected by Agilent 6,410 QQQ triple–quadruple LC mass spectrometer in a positive electrospray ionization mode. The nucleoside to base ion mass transitions of 282 to 150 (m^6^A), and 268 to 136 (A) were used to quantify the nucleosides. Quantification was performed by comparing standard curves obtained from pure nucleoside standards from the same batch of samples. The ratio of m^6^A to A was calculated according to the calibration concentration.

### caRNA or mRNA m^6^A-seq

One microliter 1:1000 diluted m^6^A spike-in from the EpiMark *N*^*6*^-Methyladenosine Enrichment Kit was added to 1 μg nonribosomal caRNA or mRNA before the fragmentation step. Then the RNA fragmentation was performed according to previously published protocols (Dominissini et al., 2012). m^6^A-IP was performed using EpiMark *N*^*6*^-Methyladenosine Enrichment Kit following the manufacturer’s protocols. Sequencing was performed at Berry Genomics (China) on an Illumina NovaSeq machine.

### Nuclear RNA-seq

Total RNA was isolated from the soluble nuclear fraction/nucleoplasm fraction of mESCs. Then the mRNA was enriched from total RNA by using the Dynabeads mRNA DIRECT™ kit. SMARTer Stranded Total RNA-seq Kit v2 (Takara) were used to prepare the library according to the manufacturer’s instructions. Sequencing was performed at the University of Chicago Genomics Facility on an Illumina NovaSeq machine.

### Nascent RNA-seq

mESCs cells were seeded and controlled to afford the same amounts of cells. After 48 h, 5-ethynyl uridine (EU) was added to 0.5 mmol/L at 60, 30, 20, and 10 min before trypsinization collection. Total RNA was purified by Trizol reagent, and nascent RNA was captured by Click-iT Nascent RNA capture Kit. ERCC RNA spike-in control (Ambion) was added to each sample (0.01 µL per sample) before constructing the library with SMARTer StarstarTotal RNA-Seq Kit V2. Sequencing was performed at the University of Chicago Genomics Facility on an Illumina NovaSeq machine.

### METTL3 and METTL14 CUT&RUN-seq

CUT&RUN was performed as described (Skene et al., 2018). Briefly, 1 × 10^7^ mESCs were harvested and washed twice with ice-cold PBS. The cells were then incubated with 2 mL nuclear isolation buffer (10 mmol/L Tris-HCl, pH = 7.5, 250 mmol/L sucrose, 400 mmol/L spermidine, 0.8% Triton X-100) and 6 mL H_2_O for 20 min on ice with frequent mixing. The pellets were collected by centrifugation at 2,500 ×*g* for 15 min. Washed cell pellet with nuclear isolation buffer and antibody buffer (20 mmol/L HEPES, pH = 7.5, 150 mmol/L KCl, 0.5 mmol/L spermidine, 2 mmol/L EDTA, 1 mg/mL BSA). The cell pellets were then incubated overnight with the antibody in Antibody buffer at 4°C. The following day, the supernatant was removed by centrifugation and the cell pellets were washed three times with wash buffer (20 mmol/L HEPES, pH = 7.5, 150 mmol/L KCl, 0.5 mmol/L spermidine). The cell pellets were then incubated with Protein A-MNase (1.5 µg in 300 µL wash buffer) for 1 h by rotation at 4°C. After three washes, the cell pellets were resuspended in 300 µL wash buffer with 6 µL 100 mmol/L CaCl_2_, mixed rapidly by inversion, and placed on ice for 30 min. The reactions were stopped by the addition of 300 µL 2× stop buffer (340 mmol/L NaCl, 20 mmol/L EDTA, 4 mmol/L EGTA, 0.05 mg/mL RNaseA, 0.05 mg/mL glycogen, 2 pg/mL spike-in DNA). The supernatant DNA was collected after centrifugation and further purified using phenol/chloroform/isoamyl alcohol (25:24:1) extractions and precipitated with 0.1 volume of 3 mol/L sodium acetate, pH = 5.2, and 3 volumes of ethanol using glycogen as carrier. Library preparation was performed by using KAPA HyperPlus Kits according to the manufacturer’s protocols. Sequencing was performed at Berry Genomics (China) on an Illumina NovaSeq machine.

### CUT&RUN-seq with spike-in

CUT&RUN was performed using CUTANA™ ChIC/CUT&RUN Kit (Epicypher) following the manufacturer’s protocols. 0.5 mol/L cells were applied for each sample with 0.2 μL SNAP-CUTANA K-MetStat Panel (H3K27me3) or 0.025 ng *E*. *coli* Spike-in DNA (METTL14). Library preparation was performed with KAPA HyperPrep Kits according to the manufacturer’s protocols. Sequencing was performed on an Illumina NextSeq 550 platform at the University of Chicago Single Cell Immunophenotyping Core.

### Histone modification ChIP-seq

mESCs were resuspended in growth media with a concentration of 10^6^ mL^−1^, cross-linked by adding 1% formaldehyde directly to the media and slowly shook at RT for 10 min. Cross-linking was stopped by adding glycine to a final concentration of 0.125 mol/L and incubating for 5 min at RT with a slow shake. The media was removed and the cells were washed twice with ice-cold PBS. Chromatin immunoprecipitation was performed using Auto iDeal ChIP-seq kit for Histone with (for *Mettl14* Control, *Mettl14* CKO, and two rescued *Mettl14* CKO mESC lines) spike-in Chromatin (Active Motif) and spike-in Antibody (Active Motif) following the manufacturer’s protocols. Library preparation was performed by using KAPA HyperPlus Kits according to the manufacturer’s protocols. Sequencing was performed at the University of Chicago Genomics Facility on an Illumina NovaSeq.

### mRNA-seq

mRNA was enriched from total RNA by using the Dynabeads mRNA purification kit. SMARTer Stranded Total RNA-seq Kit v2 were used to prepare the library according to the manufacturer’s instructions. Sequencing was performed at Berry Genomics (China) on an Illumina NovaSeq machine.

### Cell proliferation assay

500 mESCs were seeded per well in a 96-well plate. The CCK-8 Cell Counting Kit was used to detect cell proliferation in 2, 3, 4, 5, and 6 days following the manufacturer’s protocols. In brief, 10 μL CCK8 solution was added to each well and incubated for 1 h, and the absorbance at 450 nm was determined using a microplate reader (BioTek Cytation5).

### mESCs differentiation to EBs

mESCs were trypsinized to prepare single-cell suspension and 1 × 10^5^ cells were seeded in ultra-low cluster plates with DMEM supplemented with 15% FBS, 1 mmol/L l-glutamine, 0.1 mmol/L 2-mercaptoethanol, 1% nonessential amino acid. EBs were collected after seeded 7 days.

### Alkaline phosphatase staining

Cells were counted before cultured into 24-well plates (1,000 cells/well). The cells were gently rotated in dish and cultured for 5 days. The cell clones were washed twice with PBS, then the cells were fixed with 4% paraformaldehyde for 15 min at RT and stained with Alkaline Phosphatase Assay Kit. Cell colony imaging was performed by using Olympus IX73.

### co-IP with RNase treatment

Cells were washed twice by PBS, collected by scraping, and pelleted by centrifuge at 500 ×*g* for 3 min. The cell pellet was resuspended with cold lysis buffer (50 mmol/L Tris-HCl, pH = 7.5, 150 mmol/L NaCl, 1% NP40, 1:100 Protease Inhibitor Cocktail, 20 U/mL RNase inhibitor), and incubated at 4°C for 15 min with rotation. RNase A/T1 (Thermo Fisher Scientific) was added to the cell lysate. The lysate was incubated at 25°C for 10 min, and then centrifuged at 15,000 ×*g* for 15 min at 4°C. Fifty microliters of the supernatant was saved as input. The rest supernatant was incubated with 1–3 μg of specific antibodies-conjugated or IgG-conjugated Protein G Dynabeads (Thermo Fisher Scientific) at 4°C overnight. Beads were washed 5 times with the lysis buffer. Both beads and input were boiled in 1× LDS loading buffer at 95°C for 10 min and analyzed by Western blot.

### Protein *in-vitro* co-IP assay

Proteins were incubated in reaction buffer (50 mmol/L Tris-HCl, pH = 7.5, 150 mmol/L NaCl, 1:100 Protease Inhibitor Cocktail) at 25°C for 10 min. Five microliters was kept as input. The rest was incubated with 0.5 μg of specific antibodies-conjugated Protein G Dynabeads (Thermo Fisher Scientific) at RT for 45 min. Beads were washed 5 times with the wash buffer (50 mmol/L Tris-HCl, pH = 7.5, 150 mmol/L NaCl, 0.05% NP40). Beads and input were boiled with 1× LDS loading buffer at 95°C for 10 min and analyzed by Western blot.

### caRNA and mRNA m^6^A-seq data analysis

Data analysis was performed as described (Liu et al., 2020). Briefly, raw reads were trimmed with Trimmomatic-0.39 (Bolger et al., 2014) and then aligned to the mouse genome (mm10) and transcriptome (GENCODE, version M19) together with spike-in genomes including unmodified control RNA (Cypridina Luciferase) and m^6^A methylated control RNA (Gaussia Luciferase) (New England Biolabs) using HISAT (version 2.1.0) (Kim et al., 2015) with “-k 5 --rna-strandness RF” parameters. Mapped reads were separated by strands using samtools (version 1.9) (Li et al., 2009) and m^6^A peaks were called using MACS2 (version 2) (Zhang et al., 2008) with parameter “-g 1.3e8 --tsize 150 --extsize 150 --nomodel --keep-dup 5” for each strand separately. Significant peaks with *q* < 0.01 identified by MACS2 were considered. Peaks identified in at least two biological replicates were intersected using bedtools (v.2.26.0) (Quinlan and Hall, 2010) and were used in the following analysis. The number of reads mapped to mouse genome divided by number of reads mapped to m^6^A modified spike-in represented whole m^6^A level.

### Nascent RNA-seq data analysis

Data analysis was performed as described (Liu et al., 2020). Briefly, raw reads were trimmed with Trimmomatic-0.39 (Bolger et al., 2014) and then aligned to mouse genome (mm10) and transcriptome (GENCODE, version M19) together with external RNA Control Consortium (ERCC) RNA spike-in control (Thermo Fisher Scientific) using HISAT (version 2.1.0) (Kim et al., 2015) with “-k 5 --rna-strandness R” parameters. Reads on each GENCODE annotated gene were counted using HTSeq (Anders et al., 2015) and then normalized to counts per million (CPM) using edgeR (Robinson and Oshlack, 2010) packages in R. CPM was converted to attomole by linear fitting of the RNA ERCC spike-in. RNA level and EU adding time was fitted to linear equation, and the slope was estimated as transcription rate of RNA.

### Nuclear RNA-seq data analysis

Data analysis was performed as described (Liu et al., 2020). Briefly, raw reads were trimmed with Trimmomatic-0.39 (Bolger et al., 2014) and then aligned to mouse genome (mm10) and transcriptome (GENCODE, version M19) using HISAT (version 2.1.0) (Kim et al., 2015) with “-k 5 --rna-strandness RF” parameters. Reads on each GENCODE annotated gene were counted using HTSeq (Anders et al., 2015) and then differentially expressed genes (DEGs) were identified by DESeq2 (Love et al., 2014) with adjusted *P* value <0.05 and requiring at least 10 reads in at least half of the samples.

### CUT&RUN data analysis

Raw reads were trimmed with Trimmomatic-0.39 (Bolger et al., 2014) and then mapped to mouse genome (mm10) using bowtie2 (version 2.4.1) (Langmead and Salzberg, 2012) with “—dovetail” parameter in default mode: search for the multiple alignments, report the best one. Duplicated reads were removed with samtools “rmdup” (Li et al., 2009). Peaks were called using HOMER (Heinz et al., 2010) in factor style with “-F 2 -L 2 -tagThreshold 10” parameters. Peaks identified in two biological replicates were pooled using bedtools (v.2.26.0) (Quinlan and Hall, 2010) and were used in further analysis. The ENCODE blacklist (Amemiya et al., 2019) were downloaded and used to mark CUT&RUN peaks. Peaks with high intensity were identified by ROSE (Whyte et al., 2013) with default parameters.

### ChIP-seq data analysis

Raw reads were trimmed with Trimmomatic-0.39 (Bolger et al., 2014) and then mapped to mouse genome (mm10) using bowtie2 (version 2.4.1) (Langmead and Salzberg, 2012) in default mode: search for the multiple alignments, report the best one. Histone modification peaks were called using HOMER in histone mode (Heinz et al., 2010) with default parameters. Peaks identified in at least two biological replicates were intersected using bedtools (v.2.26.0) (Quinlan and Hall, 2010) and were used in further analysis.

### Clustering analysis

A matrix was built with rows as METTL3 and METTL14 CUT&RUN peaks; columns as METTL3 Control, METTL3 CKO, METTL14 Control, and METTL14 CKO mESCs samples, each with two biological replicates, around ±2.5 kb of peak centers (100 bp per bin, 8 samples × 2 replicates × 50 bins = 800 columns); and entries as METTL14 CUT&RUN signal quantified by RPKM (reads per kilobase per million mapped reads) in METTL3 Control and METTL3 CKO mESCs, or METTL3 CUT&RUN signal in METTL14 Control and METTL14 CKO mESCs. K-means clustering method was then applied with euclidean distance as measures of similarities between rows to cluster the CUT&RUN peaks into to four clusters.

### Enrichment analysis

Functional enrichment analysis was performed with DAVID (Huang et al., 2019) with default parameter.

### Statistical analysis

No statistical methods were used to predetermine sample size. At least three biological replicates were used in each experiment unless otherwise stated. Data are presented as the mean ± standard error of the mean (SEM) or standard deviation (SD). Two-tailed Student’s *t*-tests or nonparametric Wilcoxon-Mann–Whitney test (Wilcoxon rank-sum test, two-sided) were applied for calculating the *P* value indicated in the figure legends were performed to assess the statistical significance of differences between groups. Pearson correlation coefficients were calculated to assess correlation. In order to evaluate the scatter of the data points around the fitted regression line, R^2^ is used in our scatter plots which indicates the percentage of the dependent variable variation that a linear model explains. For box plots, the center line represents the median, the box limits show the upper and lower quartiles, whiskers represent 1.5× the interquartile range.

## Supplementary Material

pwad009_suppl_Supplementary_Material

## Data Availability

All data needed to evaluate the conclusions in the paper are present in the paper and/or Supplementary Materials. All sequence data have been deposited at GEO and are publicly available as of the date of publication.

## Abbreviations

caRNAs: chromatin-associated RNAs
carRNA: chromatin-associated regulatory RNA
ChIP-seq: Chromatin Immunoprecipitation Sequencing
CKO: conditional knockout
co-IP: co-immunoprecipitation
EBs: embryonic bodies
GSCs: glioblastoma stem cells
LC–MS/MS: liquid chromatography–tandem mass spectrometry
m^6^2A, *N*^*6*^: *N* ^*6*^-dimethyladenosine modification
m^6^A: *N* ^*6*^-methyladenosine
mESC: mouse embryonic stem cell
mRNA: messenger RNA
MTC: *m* ^*6*^ *A* methyltransferase complex
NPCs: neural progenitor cells
WT: wildtype.

## Code availability

This paper does not report original code.
