# Supplementary material for: METTL14 is a chromatin regulator independent of its RNA N6-methyladenosine methyltransferase activity
Source: Protein Cell. 2023 Feb 23;14(9):683–97. doi: 10.1093/procel/pwad009 (PMC10501186; doi:10.1093/procel/pwad009)
Supplement: pwad009_suppl_Supplementary_Material [file pwad009_suppl_supplementary_material.pdf]

Supplementary Materials for

METTL14 is a chromatin regulator independent of its RNA *N*^{6}-methyladenosine methyltransferase activity

Xiaoyang Dou[1,2,11], Lulu Huang[3,4,11], Yu Xiao[1,2,11], Chang Liu[1,2,5], Yini Li[6], Xinning Zhang[3,4], Lishan Yu[3,4], Ran Zhao[3,4], Lei Yang[7], Chuan Chen[7], Xianbin Yu[1,2], Boyang Gao[1,2], Meijie Qi[8], Yawei Gao[7], Bin Shen[9], Shuying Sun[6], Chuan He[1,2,10,*], Jun Liu[3,4,*]

This file includes:

Figs. S1 to S7

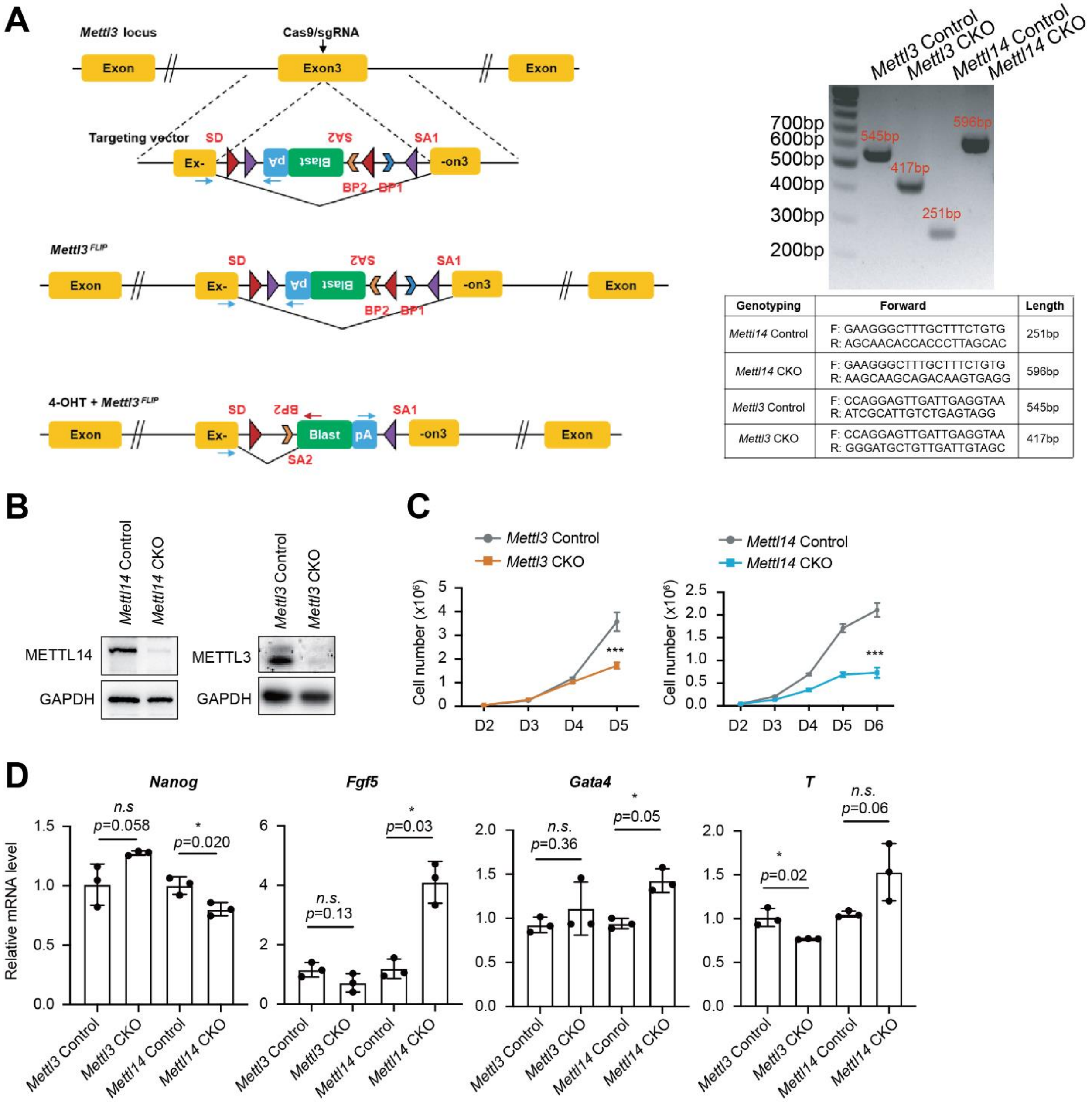

Genotyping	Forward	Length
Mettl14 Control	F: GAAGGGCTTTGCTTTCTGTG<br>R: AGCAACACCACCCTTAGCAC	251bp
Mettl14 CKO	F: GAAGGGCTTTGCTTTCTGTG<br>R: AAGCAAGCAGACAAGTGAGG	596bp
Mettl3 Control	F: CCAGGAGTTGATTGAGGTAA<br>R: ATCGCATTGTCTGAGTAGG	545bp
Mettl3 CKO	F: CCAGGAGTTGATTGAGGTAA<br>R: GGGATGCTGTTGATTGTAGC	417bp

Figure S1. METTL3 and METTL14 acted differently on stemness maintenance of mESCs. **(A)** Construction and genotyping of *Mettl3* and *Mettl14* CKO mESCs. **(B)** Immunoblot assays of METTL14 protein levels in *Mettl14* Control and *Mettl14* CKO mESCs (*left* panel), and METTL3 protein levels in *Mettl3* Control and *Mettl3* CKO mESCs (*right* panel). **(C)** Cell proliferation assay of *Mettl3* Control, *Mettl3* CKO, *Mettl14* Control, and *Mettl14* CKO mESCs. n=3 per condition. ***P< 0.001. Two-tailed Student's t-test. **(D)** Bar plot of *Nanog*, *Fgf5*, *T and Gata4* expression in *Mettl3* Control, *Mettl3* CKO, *Mettl14* Control, and *Mettl14* CKO mESCs. *P< 0.05. n = 3 biological replicates; Two-tailed Student's t-test.

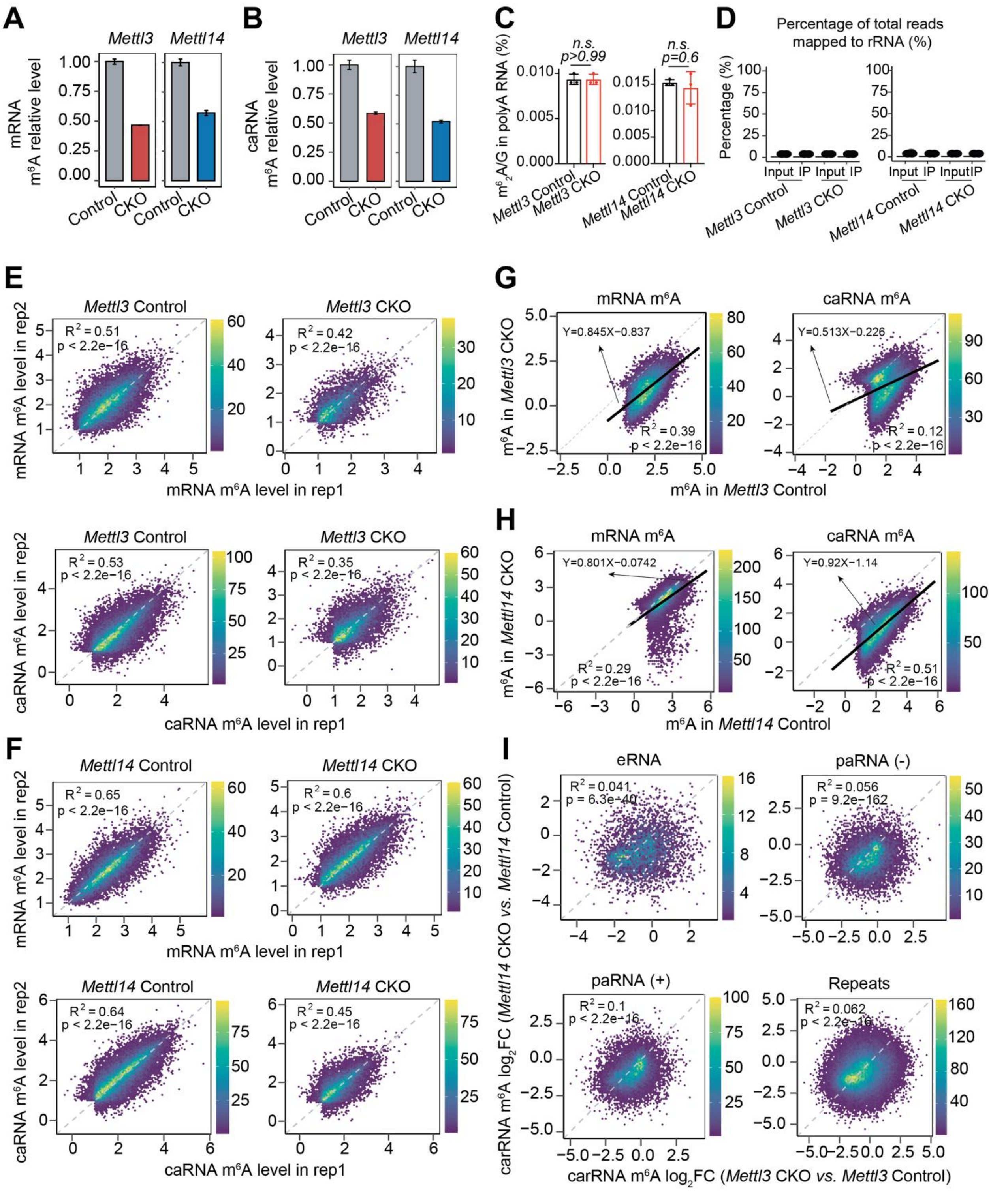

Figure S2. METTL3 and METTL14 acted similarly on m^{6}A-mediated regulation. (A-B) m^{6}A levels on mRNA (**A**) and caRNAs (**B**) in *Mettl3* Control and *Mettl3* CKO, *Mettl14* Control

and *Mettl14* CKO mESCs, respectively. m^6A level were quantified using MeRIP-seq data with the number of reads mapped to mouse genome divided by the number of reads mapped to m^6A modified spike-in. n = 2 biological replicates; error bars indicate means ± SEM. (**C**) LC-MS/MS quantification of the $m^6{}_2A$/G ratio of the PolyA-enriched RNA extracted from *Mettl3* Control, *Mettl3* CKO, *Mettl14* Control and *Mettl14* CKO mESCs. (**D**) The percentages of total reads mapped to rRNA in MeRIP-seq data of *Mettl3* Control, *Mettl3* CKO, *Mettl14* Control and *Mettl14* CKO mESCs. **(E-F)** The correlation of m^6A level on mRNA (*top* two panels) and caRNA (*bottom* two panels) between two biological replicates in *Mettl3* Control and *Mettl3* CKO (**E**), *Mettl14* Control and *Mettl14* CKO (**F**) mESCs, respectively. **(G-H)** The correlation of m^6A level on mRNA (*left* panel) and caRNA (*right* panel) between *Mettl3* Control and *Mettl3* CKO (**G**); and *Mettl14* Control and *Mettl14* CKO (**H**) in mESCs. **(I)** The correlation of m^6A fold-changes (log_2FC) on carRNAs including eRNA, sense paRNAs (paRNA(+)), antisense paRNAs (paRNA(-)) and repeats RNAs between *Mettl14* CKO *vs. Mettl14* Control and *Mettl3* CKO *vs. Mettl3* Control mESCs.

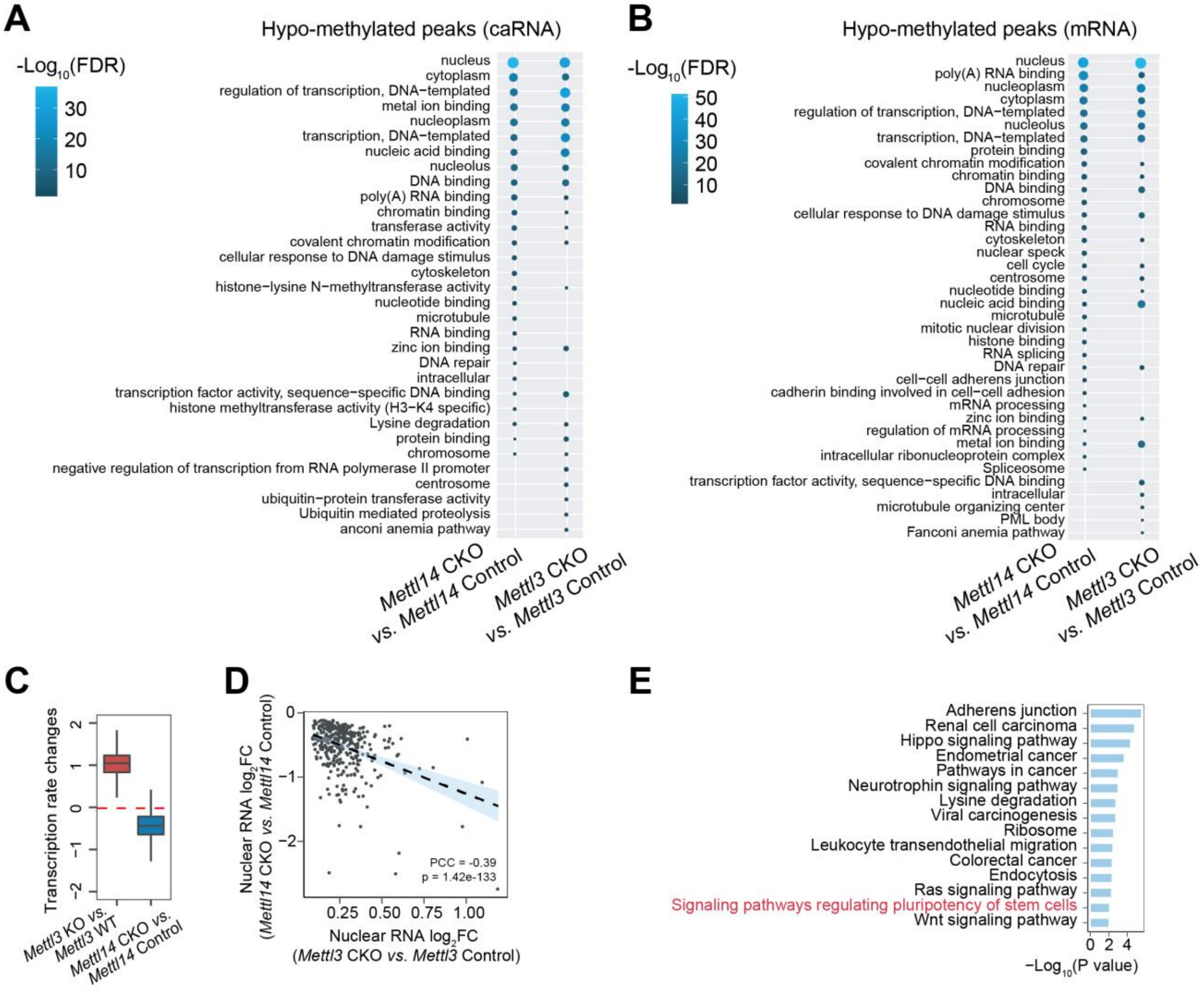

Figure S3. METTL3 and METTL14 differed in transcriptome regulation. (A-B) Enrichment analysis of genes harbored hypo-methylated peaks in caRNA (**A**) or mRNA (**B**) upon *Mettl3* knockout (**A**) or *Mettl14* knockout (**B**) in mESCs. **(C)** Boxplot of transcription rate changes upon *Mettl3* (GSE133582) or *Mettl14* knockout in mESCs. **(D)** The correlation of gene expression fold-changes (log_2FC) upon *Mettl3* or *Mettl14* knockout in mESCs. These genes are significantly (adjusted P [padj] < 0.05) up-regulated upon *Mettl3* knockout and down-regulated upon *Mettl14* knockout in mESCs. (**E**) KEGG pathway enrichment analysis of genes that are significantly (adjusted P [padj] < 0.05) up-regulated upon *Mettl3* knockout and down-regulated upon *Mettl14* knockout in mESCs.

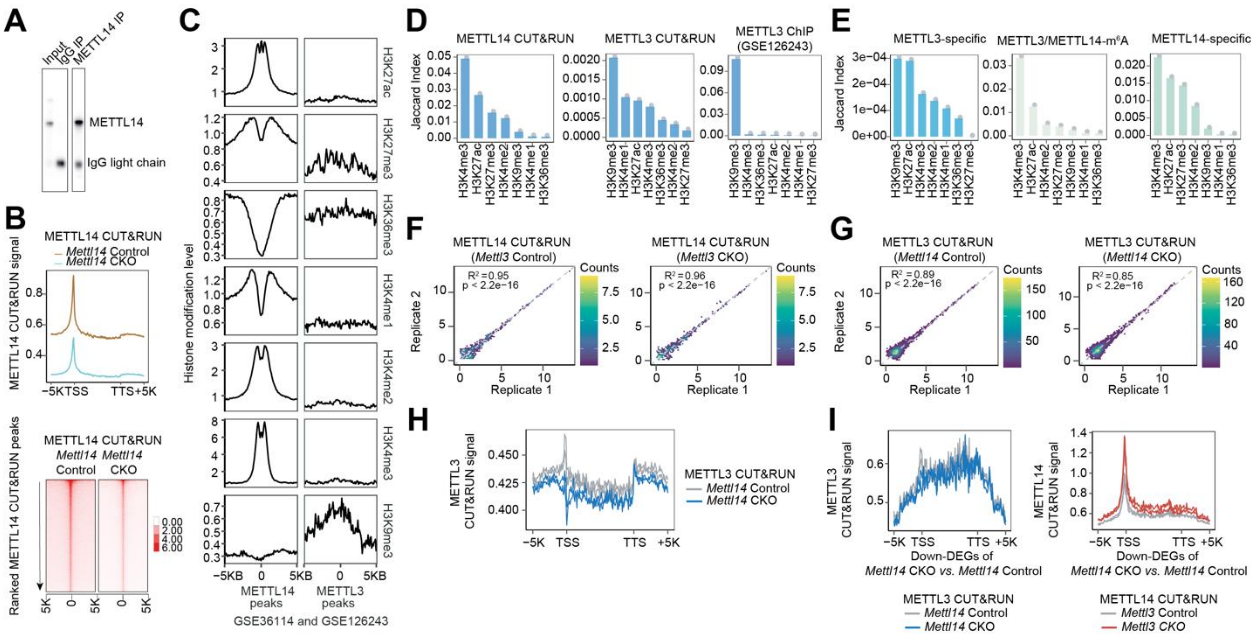

Figure S4. METTL3 and METTL14 displayed distinct chromatin binding preferences. (**A**) Western blot assays showing the specificity and IP efficiency of the METTL14 antibodies we used. (**B**) Average profiles of METTL14 at gene level (from 5KB upstream of TSS to 5KB downstream of TTS) in *Mettl14* Control or *Mettl14* CKO mESCs (*top* panel). Heatmap of METTL14 genomic binding at peaks center and their flanking regions in *Mettl14* Control or *Mettl14* CKO mESCs (*bottom* panel). CUT&RUN signal has been normalized to *E. coli* Spike-in DNAs. **(C)** Average profiles of various histone modification on METTL3 and METTL14 CUT&RUN peaks in wild type mESCs. **(D)** Bar graphs showing the overlapping ratios (Jaccard index) of METTL14 CUT&RUN, METTL3 CUT&RUN and METTL3 ChIP-seq peaks with different histone modifications (GSE36114 and GSE126243) in wild type mESCs. **(E)** Bar graphs showing the overlapping ratios (Jaccard index) of METTL14-specific sites, METTL3-specific sites and METTL3/METTL14-m^{6}A sites with different histone modifications in wild type mESCs. **(F-G)** The correlations of METTL14 CUT&RUN signal between two biological replicates in *Mettl3* Control and *Mettl3* CKO mESCs (**F**), and METTL3 CUT&RUN signal between two biological replicates in *Mettl14* Control and *Mettl14* CKO mESCs (**G**). **(H)** Average profile of METTL3 CUT&RUN signal at gene level in *Mettl14* Control and *Mettl14* CKO mESCs. **(I)** Profiles of METTL14 and METTL3 CUT&RUN signals on genes that are significantly down-regulated (down-DEGs) upon *Mettl14* knockout in mESCs.

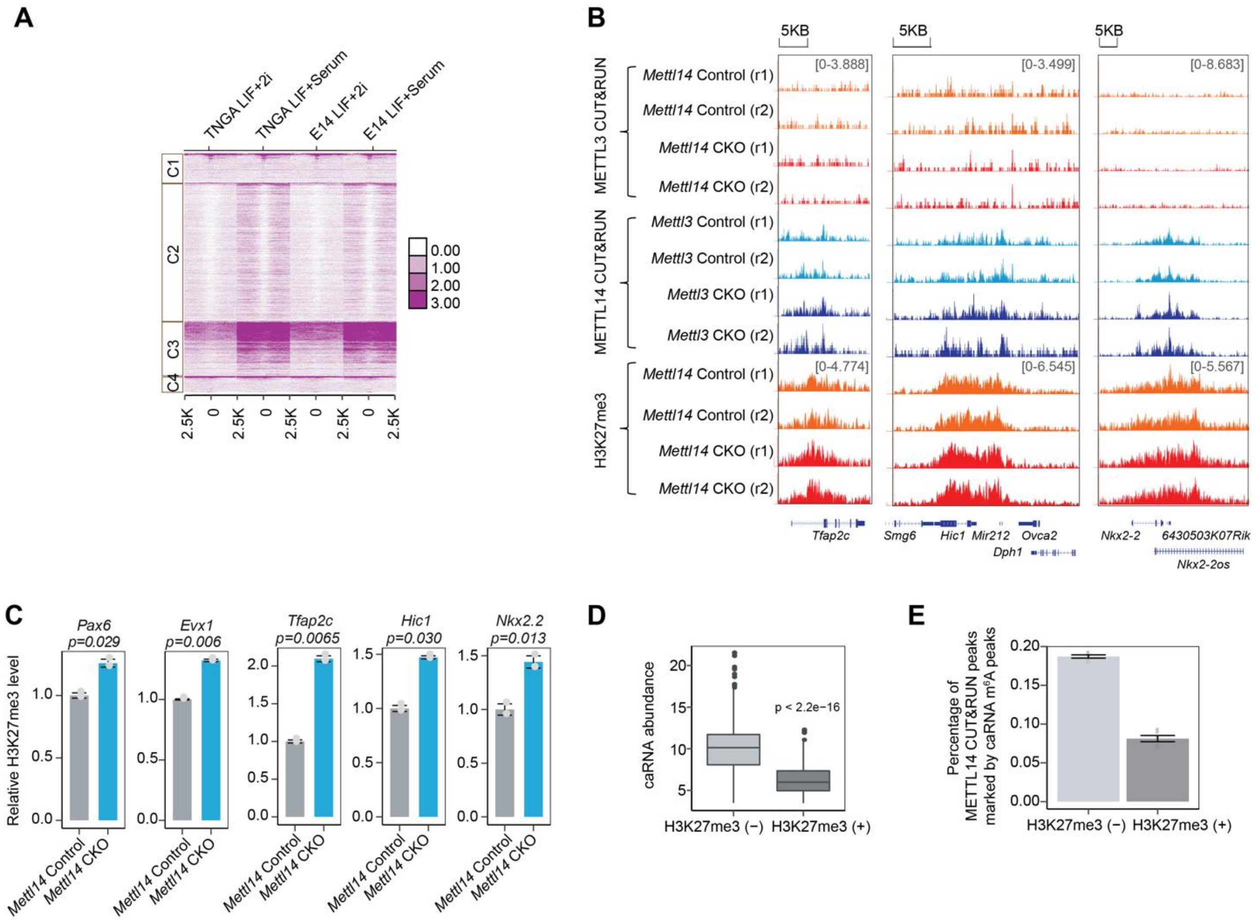

Figure S5. METTL14-occupied genomic regions marked with H3K27me3 are less enriched of m^{6}A than the non-marked ones. (**A**) Heatmap of H3K27me3 modification at four identified clusters in mESCs cultured under different cell culture conditions: "LIF+Serum" condition refers to the presence of LIF in the medium containing Serum, and "LIF+2i" condition refers to serum-free medium containing 2i. (**B-C**) IGV visualization (**B**) and bar plot for quantification (**C**) showing H3K27me3 levels on *Tfap2c*, *Hic1* and *NKx2-2* genes in *Mettl14* Control and *Mettl14* CKO mESCs. **(D)** Boxplot of caRNA abundance that derived from METTL14 bound DNA loci. METTL14 peaks were categorized into H3K27me3 modified (H3K27me3 (+)) and unmodified (H3K27me3 (-)) groups. (**E**) Bar graph showing the percentage of METTL14 peaks overlapped with caRNA m^{6}A peaks. METTL14 peaks were categorized into H3K27me3 modified (H3K27me3 (+)) and unmodified (H3K27me3 (-)) groups.

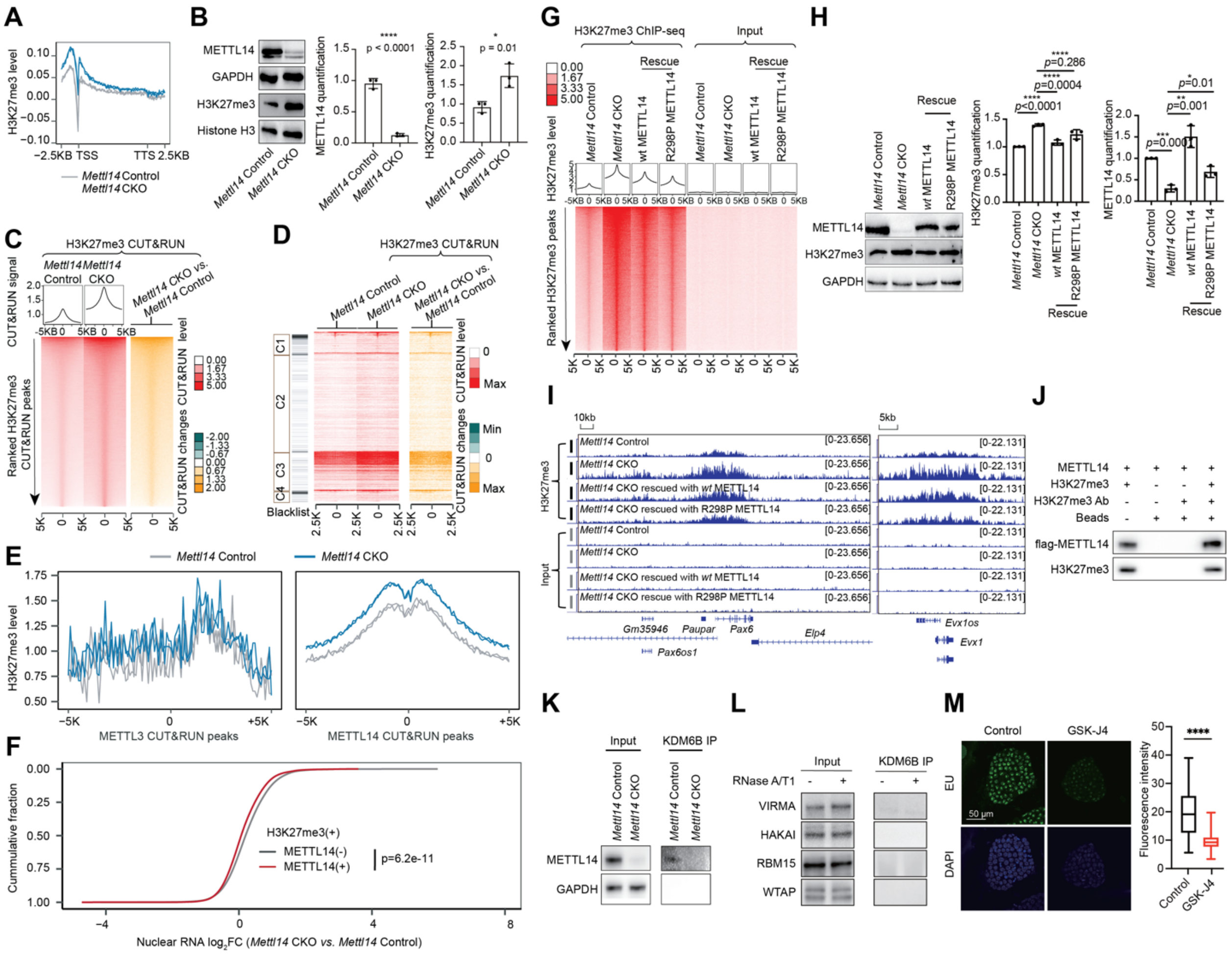

Figure S6. METTL14 directly regulates H3K27me3 deposition in mESCs. (A) Average profiles of H3K27me3 modification at gene level (from 2.5KB upstream of TSS to 2.5KB downstream of TTS) in *Mettl14* Control and *Mettl14* CKO mESCs, respectively. (**B**) Western blot (*left* panel) with quantification (*right* panel) of H3K27me3 in *Mettl14* Control and *Mettl14* CKO mESCs. n=3 per condition. P values were calculated using Two-tailed Student's t-test. (**C**) Heatmap showing H3K27me3 CUT&RUN signal (*left* panel) and H3K27me3 signal changes (*right* panel) around the peak centers (+/- 5KB, *left* panel) upon *Mettl14* CKO in mESCs. CUT&RUN signal are normalized according to SNAP-CUTANA_K-MetStat_Panel_Analysis pipeline. **(D)** Heatmap showing H3K27me3 binding and binding changes upon *Mettl14* knockout in mESCs at the four clusters of METTL3 and METTL14 peak centers and the flanking 2.5KB regions. **(E)** H3K27me3 modifications on METTL3 (*left* panel) or METTL14 (*right* panel) CUT& RUN peak centers and the flanking 5KB in *Mettl14* Control and *Mettl14* CKO mESCs, respectively. **(F)** Cumulative distributions of H3K27me3 modified gene expression fold-changes

(log_2FC) upon *Mettl14* knockout in mESCs. H3K27me3 modified genes were categorized into METTL14 bound (+) and unbound (-) groups. P values were calculated by a nonparametric Wilcoxon-Mann-Whitney test. (**G**) Heatmap showing H3K27me3 ChIP-Rx signals around the peak centers (+/- 5KB) in *Mettl14* Control, *Mettl14* CKO, and *Mettl14* CKO mESCs rescued with wild-type METTL14 or R298P mutated METTL14. ChIP-seq signal has been normalized to *Drosophila* Spike-in DNAs. (**H**) Immunoblot assays (*left* panel) with quantification (*right* panel) of METTL14 and H3K27me3 level in *Mettl14* Control, *Mettl14* CKO, *Mettl14* CKO rescued with wild-type METTL14 (*wt* METTL14) or with R298P mutated METTL14 (R298P METTL14) mESCs, respectively. (**I**) IGV profiles showing H3K27me3 modification level and its inputs around *Pax6* and *Evx1* gene loci in *Mettl14* Control, *Mettl14* CKO, and *Mettl14* CKO mESCs rescued with wild-type METTL14 or R298P mutated METTL14, respectively. (**J**) Western blots of the *in vitro* immunoprecipitated H3K27me3 peptide and its interaction with flag-METTL14. (**K**) Western blots of the immunoprecipitated KDM6B from *Mettl14* Control and *Mettl14* CKO mESCs and its interaction with METTL14. (**L**) Western blots of the immunoprecipitated KDM6B from mESCs and its interaction with the components of methyltransferase complex. (**M**) Analysis of nascent RNA synthesis in wild-type mESCs treated with DSMO and GSK-J4. Nascent RNA synthesis was detected by using a click-it RNA Alexa fluor 488 imaging kit. EU, 5-ethynyl uridine; DAPI, 4′, 6-diamidino-2-phenylindole.

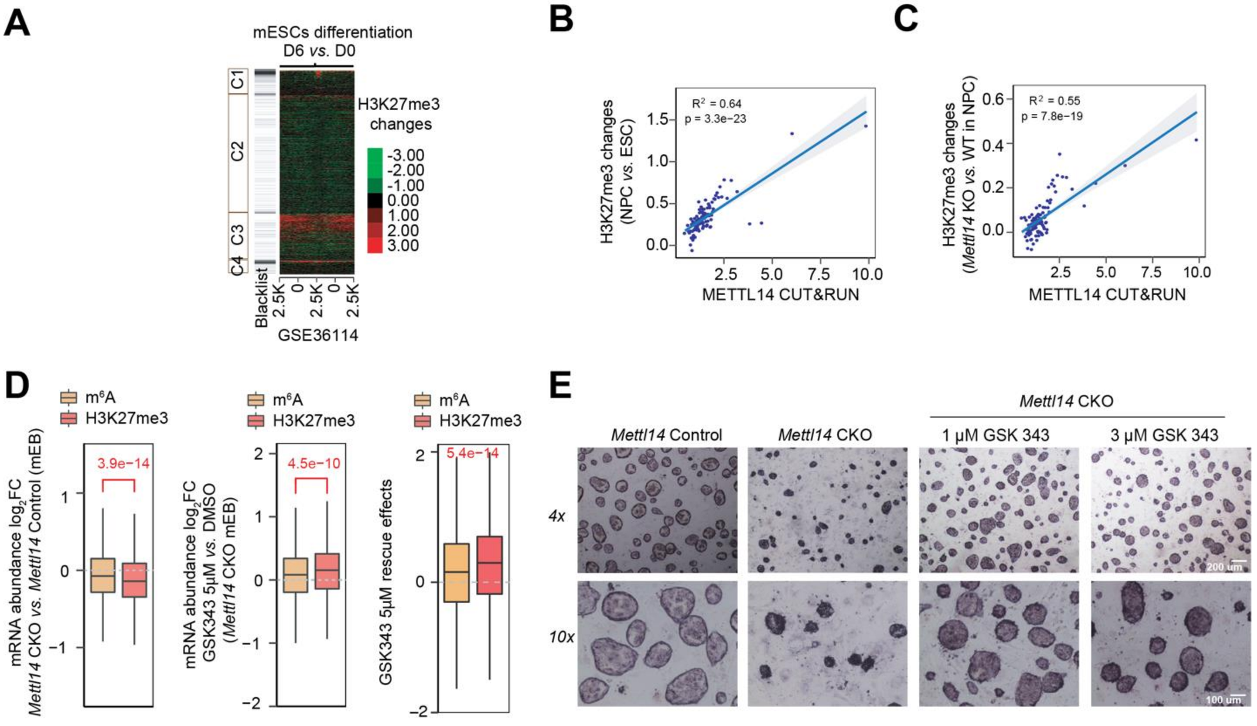

Figure S7. METTL14 regulates H3K27me3 changes during neuronal differentiation of mESCs. (A) Heatmap showing H3K27me3 modification level changes during mESCs differentiation (day 6 *vs.* day 0) on four clusters of METTL3 and METTL14 CUT&RUN peak centers and the flanking 2.5KB regions. (**B-C**) Scatter plot showing the correlation between METTL14 CUT&RUN signal and H3K27me3 level changes comparing NPCs *vs.* ESCs (**B**), and *Mettl14* KO *vs.* wildtype (WT) NPC (**C**). Sites ranked by METTL14 CUT&RUN signal were grouped and averaged into 100 data point. (**D**) Boxplot of gene expression level fold-changes (log_2FC) upon *Mettl14* knockout (*left* panel); after GSK343 treatment *in Mettl14* knockout mEB (*middle* panel); or the rescue degree (*right* panel). Rescue degree was calculated as log_2FC from (*middle* panel) subtracted the log_2FC from (*left* panel). Genes were categorized into two subgroups according to whether they were modified by m^6A or H3K27me3. P values were calculated by a nonparametric Wilcoxon-Mann-Whitney test. (**E**) Alkaline phosphatase staining of *Mettl14* Control, *Mettl14* CKO, and *Mettl14* CKO mESCs treated with GSK343 under two different concentrations.
